# Genome wide DNA methylation analysis identifies novel molecular subgroups and predicts survival in neuroblastoma

**DOI:** 10.1038/s41416-022-01988-z

**Published:** 2022-09-29

**Authors:** H. Lalchungnunga, Wen Hao, John M. Maris, Shahab Asgharzadeh, Kai-Oliver Henrich, Frank Westermann, Deborah A. Tweddle, Edward C. Schwalbe, Gordon Strathdee

**Affiliations:** 1 Biosciences Institute, Newcastle University Centre for Cancer, Newcastle University, Newcastle, UK; 2 Division of Oncology and Center for Childhood Cancer Research, Children’s Hospital of Philadelphia, Philadelphia, PA USA; 3 Children’s Hospital Los Angeles, The Saban Research Institute and Keck School of Medicine, University of Southern California, Los Angeles, CA USA; 4 Hopp Children’s Cancer Center Heidelberg (KiTZ), Heidelberg, Germany; 5 Division of Neuroblastoma Genomics, German Cancer Research Center (DKFZ), Heidelberg, Germany; 6 Translational and Clinical Research Institute, Newcastle University Centre for Cancer, Newcastle University, Newcastle, UK; 7 Department of Applied Sciences, Northumbria University, Newcastle, UK

**Keywords:** Paediatric cancer, Cancer epigenetics

## Abstract

**Background:**

Neuroblastoma is the most common malignancy in infancy, accounting for 15% of childhood cancer deaths. Outcome for the high-risk disease remains poor. DNA-methylation patterns are significantly altered in all cancer types and can be utilised for disease stratification.

**Methods:**

Genome-wide DNA methylation (*n* = 223), gene expression (*n* = 130), genetic/clinical data (*n* = 213), whole-exome sequencing (*n* = 130) was derived from the TARGET study. Methylation data were derived from HumanMethylation450 BeadChip arrays. t-SNE was used for the segregation of molecular subgroups. A separate validation cohort of 105 cases was studied.

**Results:**

Five distinct neuroblastoma molecular subgroups were identified, based on genome-wide DNA-methylation patterns, with unique features in each, including three subgroups associated with known prognostic features and two novel subgroups. As expected, Cluster-4 (infant diagnosis) had significantly better 5-year progression-free survival (PFS) than the four other clusters. However, in addition, the molecular subgrouping identified multiple patient subsets with highly increased risk, most notably infant patients that do not map to Cluster-4 (PFS 50% vs 80% for Cluster-4 infants, *P* = 0.005), and allowed identification of subgroup-specific methylation differences that may reflect important biological differences within neuroblastoma.

**Conclusions:**

Methylation-based clustering of neuroblastoma reveals novel molecular subgroups, with distinct molecular/clinical characteristics and identifies a subgroup of higher-risk infant patients.

## Introduction

Neuroblastoma is the most common extracranial solid cancer in infants and children [1, 2]. It is responsible for 8% of paediatric cancer cases, but 15% of cancer deaths in children. While outcome for low-risk patients is excellent, with 5-year PFS survival >85%, outcome in high-risk cases and in older children remains poor with survival of <50% [3]. Features that are known to be associated with poor outcome include age >18 months [4] and the presence of recurrent genetic abnormalities, such as *MYCN* amplification [5] and chromosome 11q deletion [6].

Neuroblastoma has been classified into risk groups using multiple approaches, including the International Neuroblastoma Staging System (INSS), Children’s Oncology Group risk stratification (incorporating INSS and other clinical/genetic features, such as age of diagnosis and *MYCN* amplification) and the consensus International Neuroblastoma Risk group (INRG) classification system (which incorporates a pre-surgical staging system based on tumour imaging as opposed to surgical resection, as used in INSS) [3].

DNA methylation is the most commonly occurring covalent modification of DNA in humans and is a key epigenetic mechanism that plays important roles in the regulation of gene expression [7, 8]. As with other types of cancer, neuroblastoma is characterised by widespread alterations in DNA methylation, including global hypomethylation alongside localised increases in methylation at promoter-associated CpG islands. A number of studies have identified potentially important tumour suppressor genes that are frequently inactivated due to promoter hypermethylation [9, 10]. Furthermore, changes in genome-wide DNA-methylation patterns have been found to be useful for sub-classification and identifying different molecular subgroups in other types of malignancy, such as medulloblastoma, breast and ovarian cancer [11–13]. Identification of such subgroups can be a key step in allowing the rational development of targeted therapies [14]. Initial molecular analysis of neuroblastoma by Henrich et al. [15] and Olsson et al. [16], using unsupervised hierarchical clustering has suggested that there may be 2–3 DNA-methylation-dependent subgroups, which showed clear overlaps with specific genetic and clinical disease features. However, more recently developed dimensional reduction techniques, such as t-distributed stochastic neighbour embedding (t-SNE) [17, 18], have been shown to be useful for a more refined identification of distinct molecular subgroups.

In this report, we used DNA-methylation data collected from 223 primary neuroblastoma samples as part of the Therapeutically Applicable Research To Generate Effective Treatments (TARGET) initiative. t-SNE-based sub-clustering identified five robust, methylation-based clusters, which were validated in an independent cohort. Three of the clusters were associated with known genetic features and two have not previously been reported. These novel clusters exhibit differential survival and can be used to identify subsets of patients, particularly infants with higher-risk disease.

## Materials and methods

### Datasets used in the study

DNA methylation for primary neuroblastoma samples used in the study was obtained from publicly available data deposited as part of the Therapeutically Applicable Research To Generate Effective Treatments (TARGET) initiative (https://ocg.cancer.gov/programs/target). Genome-wide DNA methylation was assessed using Illumina Infinium HumanMethylation450 (450 k) array. Clinical and molecular data were available for 213 samples, summarised in Table 1. This sample set primarily focussed on high-risk diseases, where there is the most unmet clinical need.

**Table 1:** Study cohort; clinical and molecular details of the test dataset.

Cohort size	223 (no clinical information for 10 samples)
Age at diagnosis in year	
Median (min–max)	2.8 (0–20)
Sex	
M	127
F	86
M:F ratio	1.47:1
*MYCN* status	
Amplified	50
Non-amplified	163
Survival information	
Median event-free survival in year (min–max)	2.3 (0–13.5)
INSS stage	
Stage-1	15
Stage 2b	1
Stage 3	6
Stage-4	167
Stage-4s	24
COG risk	
High risk	168
Intermediate risk	14
Low risk	31
Histology (INPC classification)	
Favourable	47
Unfavourable	150
Unknown	16
Ploidy	
Diploid	78
Hyperdiploid	132
Unknown	3

Paired mutational and transcriptomic data were available for 130 samples, generated using whole genome and whole-exome sequencing and RNA sequencing [19, 20], and deposited in the TARGET database (https://ocg.cancer.gov/programs/target/projects/neuroblastoma).

DNA-methylation data from an additional unselected sample set of 105 patients, which has been previously reported [15] was also used for validation of the methylation clusters. This data was also obtained from Illumina Infinium HumanMethylation450 arrays. Clinical details for this cohort are in Supplementary Table 1. A flowchart detailing the numbers of samples for which specific types of data were available is shown in Supplementary Fig. 1.

### Bioinformatic analysis

#### DNA-methylation processing

We obtained raw methylation array data (idat format). Array quality check, normalisation and processing were performed using the R package 'Minfi' [21] to filter out poorly performing probes that may have a confounding effect on downstream analyses. Briefly, array background fluorescent signal bias correction was performed using ‘noob (normal-exponential out-of-band)’ [22], probes that had detection *P* value >0.01 in 50% of samples were removed, cross-reactive probes [23] were also removed, as were probes from X and Y chromosomes and, probes with proximal SNPs with a minor allele frequency of 5% or greater and maximum distance (from CpG to SNP) of two bases. This resulted in the retention of 426,682 probes. We extracted the methylation score (β-value) for the remaining probes, which can range from 0 to 1, indicating unmethylated and full methylation status, respectively.

### Unsupervised clustering and subgroup identification

We selected the 10,000 most variable probes by the standard deviation for clustering and subgroup identification. We calculated the pairwise distance matrix using ‘1 − weighted Pearson correlation coefficient’ using ‘weights’ R package (V.1.0) as previously described [18, 24]. We then employed the resulting distance matrix to perform the t-distributed stochastic neighbour embedding (t-SNE, v0.13). The following parameters were used to perform t-SNE: theta = 0, max_iter = 5000, perplexity = 30, pca = FALSE, is_distance = T. We performed consensus clustering by randomly sampling 80% of data, mapped back the result to the full dataset and the process was repeated 256 times. At each iteration, dimension reduction to two and three dimensions were calculated using the t-SNE and for each dimension, between 2 and 10 clusters were assigned using k-means clustering. We then calculated the average modal score for cluster assignment for each combination of dimension and cluster as previously described [11]. In addition, we measured the average silhouette score for multiple clusters (ranging from 1 to 10) to select the optimal dimension and cluster for discrimination of robust molecular subgroups.

### Detection of copy number alterations

DNA-methylation-derived copy number alterations were assessed using the conumee R package (v.0.13). Probes on X and Y chromosomes were retained for assessment of copy number alteration. Briefly, the combined intensities of both methylated and unmethylated signals were normalised against a set of normal adrenal samples (*n* = 10) (https://ocg.cancer.gov/programs/target/projects/neuroblastoma), and probes within predefined genomic domains were then combined resulting in a bin of minimum size of bases and minimum number of probes. We selected default parameters, minimum size (50,000 bases) and minimum number of probes (15 probes) for the analysis. Data generated using conumee were then utilised to generate copy number variation heatmaps for each sample across all chromosomes, as previously described [25]. Chi-squared tests were used to test subgroup specificity of copy number alterations.

### Identification of differentially methylated regions for individual methylation clusters

We identified differentially methylated region (DMR) candidates specific for individual methylation clusters using DMRcate [25]. DMRs were selected on the basis of an average beta-value difference across the DMR that exceeded 0.3. Gene ontology analysis was performed using the GOregion function in missMethyl R package [26], as this package accounts for bias in CpG representation for individual genes on the 450 K array. Gene lists for this analysis were generated by linking the identified DMRs to the nearest annotated gene (DMRs not associated with a known gene or >20 kb from a known gene were not included in this analysis to increase the likelihood that differential methylation was linked to altered gene function).

### Gene expression dataset

Gene expression data, generated by RNA-seq, was available for 130 paired samples [21] obtained from the TARGET website. Correlation between the methylation status of cg11625005 probe located within the *TERT* promoter and its corresponding gene expression was assessed using Spearman’s correlation method.

### Visualisation of mutation data

Mutation data were visualised with an Oncoplot, drawn using maftools (v.2.4.05). Cluster-specific enrichment for *ALK* gene mutation was assessed using the Chi-squared test. The *TTN* and *MUC16* genes were excluded from this analysis, as their large size results in a high frequency of passenger mutations [27].

### Survival analysis

Survival information was available for 195 patients from the original cohort and 105 samples from the second cohort. We performed progression-free survival (PFS) analysis on both sets of data separately using the Kaplan–Meier method. The survival of subgroups was compared using log-rank test. Multivariable analysis was performed with the Cox-proportional Hazards model using IBM SPSS statistics 27 (IBM, NY, USA).

The significant threshold for all statistical test was *P* < 0.05. Unless otherwise stated, all the bioinformatic and statistical tests (other than multivariable survival analysis) were performed using R (version 3.5.3).

## Results

### Unsupervised clustering of primary neuroblastoma identifies five DNA-methylation-based subgroups

**Fig. 1:**
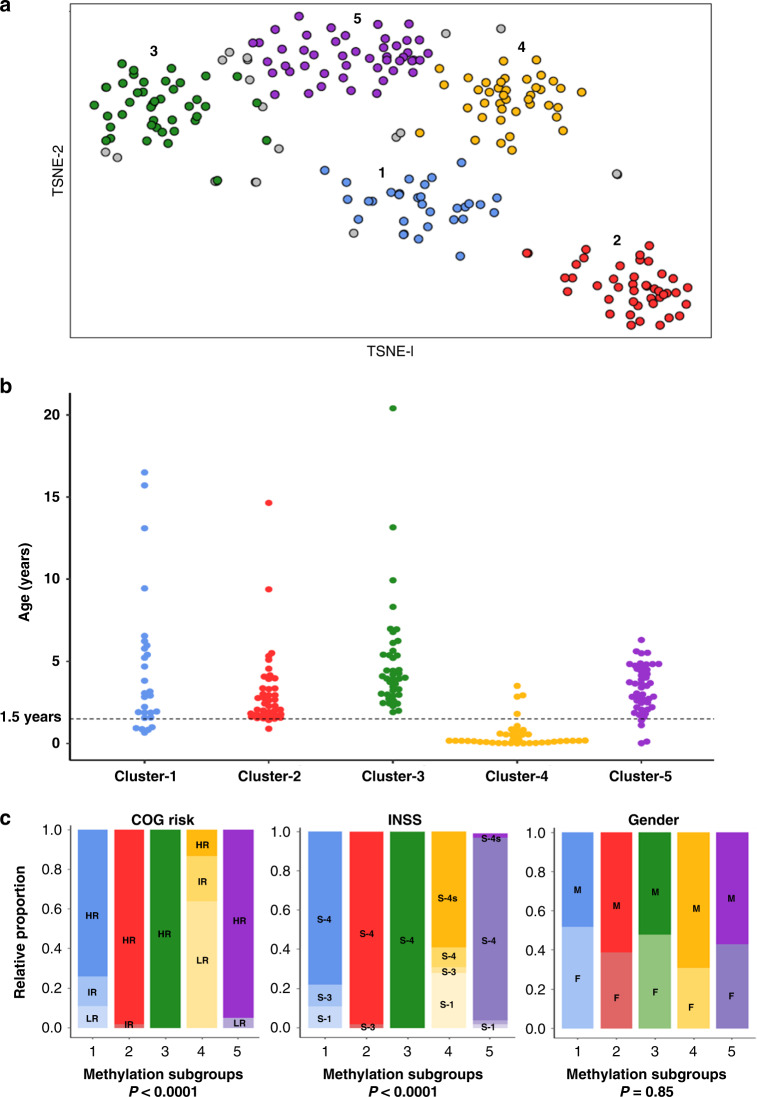
Methylation-based identification of molecular subgroups in neuroblastoma. **a** Unsupervised consensus clustering using t-SNE identifies five clusters. Grey colour indicates low confidence samples (*n* = 18), having below 70% modal cluster assignment by resampling of samples. Number of sample mapping to clusters—Cluster-1 (*n* = 32), Cluster-2 (*n* = 43), Cluster-3 (*n* = 42), Cluster-4 (*n* = 40) and Cluster-5 (*n* = 47). **b** Age distribution at diagnosis across the clusters. Clinical information was available for 195/204 classifiable samples (*n* = 28, 41, 41, 39 and 46 for Clusters 1–5, respectively). The dotted horizontal line indicates 1.5 years at diagnosis. As shown, the majority, but not all cases diagnosed below 1.5 years are present in Cluster-4. **c** Cluster distribution for gender (M—male, F—female), COG risk (HR— high risk, IR—intermediate risk, LR—low risk), INSS (S1—stage-1, S3—stage 3, S4—stage 4, S-4s—stage 4 s).

Neuroblastoma is associated with a number of recurrent genetic defects, but lacks clearly defined molecular subgroups. Here, we have used genome-wide DNA-methylation data from 223 predominantly high-risk neuroblastoma patients to assess whether neuroblastoma can be stratified into methylation-based subgroups. Using a consensus clustering, t-SNE/k-means method, we identified five robust molecular subgroups (Fig. 1a). Cluster reproducibility was higher when t-SNE derived two rather than three dimensions (Supplementary Fig. 2A). In addition, we tested the optimal number of clusters by comparing the silhouette score (measuring the quality of clusters) for different number clusters (1–10). The combination of number of dimensions (two) and clusters (five), that have the highest reproducibility and the highest silhouette scores, was selected (Supplementary Fig. 2). In total, 204/223 (91%) samples could be reproducibly assigned to clusters, demonstrated by a positive silhouette score and with consistent cluster assignment in >70% of iterative replicates (hereinafter referred to as methylation classifiable samples). A subset of samples (*n* = 18), that could not be assigned reproducibly to clusters or had a negative silhouette score (*n* = 1) were designated as non-classifiable samples and were not considered for further analyses, as including samples with uncertain cluster designations may have limited subsequent studies of the biological basis of the clusters. Illustrative hypomethylation and hypermethylation events associated with the subgroups are shown in Supplementary Fig. 3.

### Clusters have distinct clinical and genetic features

The genetic and clinical information available for samples under study are summarised in Table 1. We investigated subgroup specificity for multiple clinical features, including age, gender, Children’s Oncology Groups risk classification (COG), and the International Neuroblastoma Staging System (INSS). We found Cluster-4 to be predominantly infant with age <1.5 years at diagnosis (35/39, 90%, 1 unknown, *χ*^2^ test, *P* < 0.0001; Fig. 1b). Infant cases were much rarer in other clusters (11/156, 8 samples of unknown age), although this still represented about a quarter of infant cases that did not map to the main infant cluster.

Cluster-4 was predominantly enriched in low-risk (25/39, 64%, 1 sample unknown) and intermediate-risk (9/39, 23%) tumours according to Children’s Oncology Group (COG) risk stratification, whereas all other subgroups were found to be predominantly high-risk (146/156, 94%, *χ*^2^ test, *P* < 0.0001, 8 samples unknown; Fig. 1c). While most non-Cluster-4 samples were stage-4 (146/156, 94%, 8 samples unknown) according to the International Neuroblastoma Staging System (INSS), Cluster-4 samples were predominantly stage-4s and stage-1 [23/39 (59%) and 11/39 (28%), respectively, *χ*^2^ test, *P* < 0.0001, 1 sample unknown]. We did not find significant association between the clusters and gender.

### Clusters showed distinct genetic and cytogenetic profiles

**Fig. 2:**
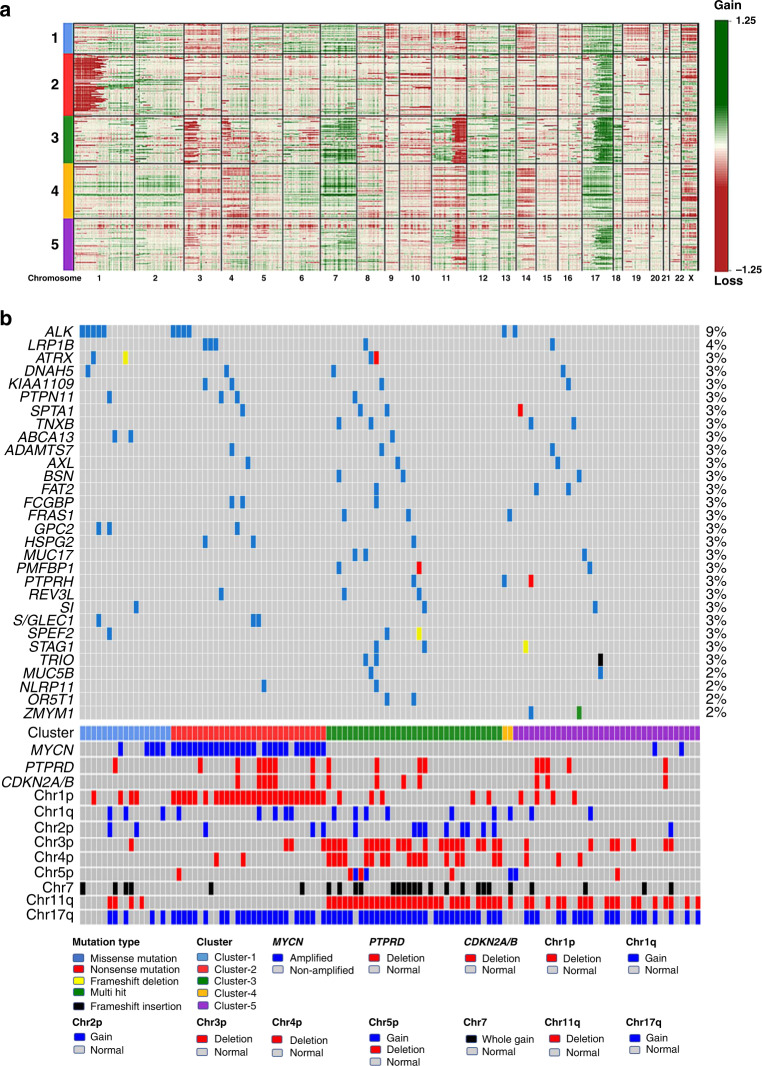
Methylation subgroups have distinct cytogenetic profiles. **a** Heatmap for copy numbers across all chromosomes illustrates subgroup-specific cytogenetic aberrations. Red colour indicates loss of focal region/chromosomal arm, green indicates gain of focal region/chromosomal arm. Cluster designations (1–5) are indicated at the left-hand side and chromosome number is listed at the bottom of the figure. Number of samples—Cluster-1 (*n* = 32), Cluster-2 (*n* = 43), Cluster-3 (*n* = 42), Cluster-4 (*n* = 40) and Cluster-5 (*n* = 47). **b** Oncoplot showing genetic mutation data across the methylation clusters. The presence or absence of mutation for the top 30 frequently mutated genes are indicated by a filled (mutated) or unfilled (unmutated) box for 116 paired methylation classifiable samples, with the type of mutation indicated by the fill colour, as shown. Samples are ordered by their methylation cluster designation, and the presence or absence of specific chromosomal aberrations and gene-specific amplification/deletion are also indicated below. Number of sample distributed across clusters (*n* = 17, 29, 33, 2 and 35 for Clusters 1–5, respectively).

From the methylation data, we inferred chromosomal aberrations (deletions or gains) covering all or part of chromosomal arms and examined specific loci previously implicated in neuroblastoma (*MYCN*, *TP53, TERT*) using conumee analysis [23]. *MYCN* amplification data, derived from FISH analysis, was already available for all samples (Table 1). Conumee analysis confirmed all 50 cases with *MYCN* amplification and identified one additional case with amplification from the remaining 163 cases. Data obtained from this analysis are illustrated in Fig. 2a and the results are summarised in Table 2. Representative example copy number plots are shown for each cluster in Supplementary Fig. 4A–E.

**Table 2:** Summary of clinical, molecular and cytogenetic profiles for methylation-derived subgroups.

Cluster no.	1	2	3	4	5	*P* value^a^
No. samples	32	43	42	40	47	NA
Age						
Infant	5 (19%)	2 (5%)	0 (0%)	**35 (90%)**	4 (9%)	<0.0001
Children	22 (81%)	39 (95%)	42 (100%)	4 (10%)	42 (91%)	
Unknown	4	2	1	1	1	
Chr1p−	7 (22%)	**38 (88%)**	7 (17%)	2 (5%)	5 (11%)	<0.0001
Chr1q+	6 (19%)	9 (21)	7 (17%)	2 (5%)	4 (8%)	0.05
Chr2p+	4 (13%)	4 (10%)	**11 (26%)**	0 (0%)	1 (2%)	0.0003
Chr3p−	2 (6%)	3 (7%)	**29 (69%)**	3 (8%)	9 (19%)	<0.0001
Chr4p−	0 (0%)	2 (5%)	**21 (50%)**	2 (5%)	4 (8%)	<0.0001
Chr5p+/−	0 (0%)	3 (7%)	5 (12%)	3 (8%)	4 (9%)	0.65
Chr11q−	6 (19%)	1 (2%)	**40 (95%)**	1 (3%)	**24 (51%)**	<0.0001
Chr17q+	6 (19%)	**33 (77%)**	**38 (90%)**	4 (10%)	**25 (53%)**	<0.0001
* MYCN* amp	5 (16%)	**39 (91%)**	0 (0%)	0 (0%)	3 (6%)	<0.0001
* PTPRD*−	3 (9%)	**13 (30%)**	6 (14%)	3 (8%)	6 (13%)	0.03
* CNKN2A/B*−	1 (3%)	**12 (28%)**	8 (19%)	4 (10%)	5 (11%)	0.02
* TERT* amp	1	1	4	1	1	0.3
Whole chromosomal gains or losses						
Chr7+	8 (25%)	3 (7%)	**20 (48%)**	8 (17%)	4 (9%)	<0.0001
At least 1	**27 (84%)**	9 (21%)	**21 (50%)**	**27 (68%)**	13 (28%)	<0.0001
2 or more	**15 (53%)**	4 (12%)	6 (14%)	**20 (50%)**	8 (17%)	<0.0001
NC	19					

^a^*P* values derived from Chi-square tests. The bold numbers indicate specific enrichment. NC represents non-classifiable samples.

In all, 91% (39/43) of Cluster-2 samples were *MYCN-*amplified compared with 5% (8/161) of samples from the other clusters (*χ*^2^ test, *P* < 0.0001). This cluster was also strongly associated with 1p-del (38/43, 88%, *χ*^2^ test *P* < 0.0001), and exhibited significantly higher levels of *PTPRD* and *CDKN2A/B* deletion compared with other clusters (Table 2).

Cluster-3 was significantly associated with 11q deletion (40/42 samples, 95%, *χ*^2^ test, *P* < 0.0001). Additionally, Cluster-3 had significant enrichment for the gain of whole chromosome 7 [20/42 (48%) vs 23/162 (14%) in other clusters, *χ*^2^ test, *P* < 0.0001], while gain of other whole chromosomes was rare in this cluster (Table 2). This cluster was also associated with increased levels of multiple other typical segmental chromosomal loss and gains (SCA), including 2p-gain, 3p-deletion and 4p-deletion, summarised in Table 2.

Cluster-4 patients were predominantly infants (35/39, 90%, 1 unknown), and this cluster was found to have significant enrichment for whole chromosomal gains or losses (WCA), particularly for abnormalities in two or more whole chromosomal changes [20/40 (50%), *χ*^2^ test, *P* < 0.0001] as shown in Table 2.

Cluster-1 and Cluster-5 represent novel subgroups. Cluster-1 was not specifically enriched for recurrent genetic/cytogenetic or clinical features that have been previously reported to be associated with poor outcome. However, this cluster did show high levels of WCA, which were found in 27/32 (84%, *χ*^2^ test, *P* < 0.0001]) (Table 2). In addition, there were low levels of many of the commonly occurring abnormalities in neuroblastoma that were predominantly associated with Clusters 2–4, including *MYCN* amplification (16%), infant diagnosis (19%) and 11q deletion (19%). Overall, this cluster appears to represent a novel neuroblastoma molecular subgroup that is defined by enrichment for WCA in non-infant patients.

Cluster-5 lacks a single unifying molecular feature. It does exhibit enrichment of 11q deletion (24/47 cases, 51%, *χ*^2^ test, *P* < 0.0001). However, this incidence is far less prevalent than in Cluster-3 (51% vs 95%). Furthermore, Cluster-5 samples were also less likely than those in Cluster-3 to have associated deletions of chromosome 3p (19% vs 69%) or 4p (8% vs 50%) and showed no association with a gain of whole chromosome 7 as seen in Cluster-3 (Table 2). Thus, Cluster-5 appears to represent a second novel molecular subgroup which exhibits lower levels of known recurrent genetic changes.

### Cluster-mutational profiles

We investigated the possibility that these clusters could be also driven by different mutational profiles. Mutation data, derived from whole genome and whole-exome sequencing [18] was available for 130 samples, from which 116 were paired methylation classifiable samples (all in children, age at diagnosed >1.5 years). Mutation profiling was only available for non-infant neuroblastoma patients, resulting in very limited representation of infant/Cluster-4. As previously reported for neuroblastoma [18], the mutation frequencies were generally low, with *ALK* (9%), *PTPN11* (3%) and *ATRX* (3%) mutations being the most common. As shown in Fig. 2b, mutation frequencies were generally similar across all four non-infant clusters, although there was preliminary evidence that *ALK* mutation may be preferentially associated with the novel Cluster-1 [5/17 (29%) vs 6/99 (6%) samples mutated, *P* = 0.03).

### *TERT* expression is correlated with Cluster-2 and with methylation in non-cluster-2 samples

**Fig. 3:**
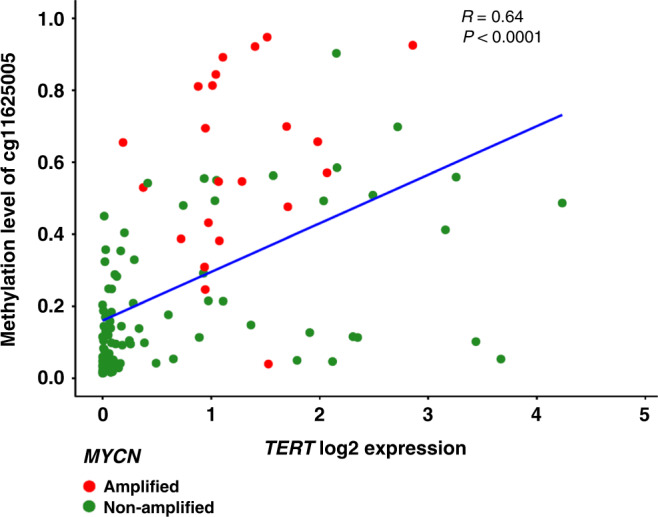
*TERT* methylation and expression are correlated in neuroblastoma. *TERT* gene expression was assessed using RNA-seq in 120 paired methylation classifiable samples. *MYCN*-amplified cases (*n* = 23) of neuroblastoma showed higher expression of *TERT* compared to the non-amplified cases (*n* = 97, *P* = 0.001). In addition, the *MYCN*-amplified cases also showed higher levels of DNA methylation at *TERT* promoter CpG site cg11625005, as compared to non-amplified cases (*P* < 0.0001). Furthermore, we found a strong correlation between *TERT* promoter methylation and gene expression, assessed using Spearman correlation (*r* = 0.62, *P* < 0.0001). Line represents linear regression of methylation vs expression in *TERT*. Red colour represents *MYCN*-amplified cases, and green represents non-amplified cases.

*TERT* expression has been suggested to be a key molecular feature in neuroblastoma, and increased *TERT* expression has been associated with *MYCN* amplification [28]. Consistent with this, analysis of expression of the *TERT* gene demonstrated that *TERT* expression was significantly higher in the Cluster-2/*MYCN*-amplified subgroup (Supplementary Fig. 6). *TERT* gene expression has also been linked to methylation of the specific CpG site cg11625005 (chr5:1,295,737 in hg19) in the *TERT* promoter in neuroblastoma and other tumour types [28, 29]. Therefore, we assessed *TERT* expression and cg11625005 methylation across the cohort. We found higher methylation levels of cg11625005 in Cluster-2/*MYCN*-amplified samples as compared to other clusters (median beta-value 0.56 vs 0.11, *P* < 0.0001, Fig. 3). However, we also found a relatively low correlation between cg11625005 and *TERT* expression in Cluster-2 (*r* = 0.36, *P* = 0.09, Supplementary Fig. 5A). In contrast, while average methylation levels were lower in non-Cluster-2 samples, there was a stronger, statistically significant, correlation between cg11625005 methylation and expression (*r* = 0.5, *P* < 0.0001), suggesting methylation of this CpG site might be primarily of relevance in neuroblastoma that lacks *MYCN* amplification (Supplementary Fig. 5B).

### Survival analysis in the molecular subgroups identifies novel associations with patient outcome

**Fig. 4:**
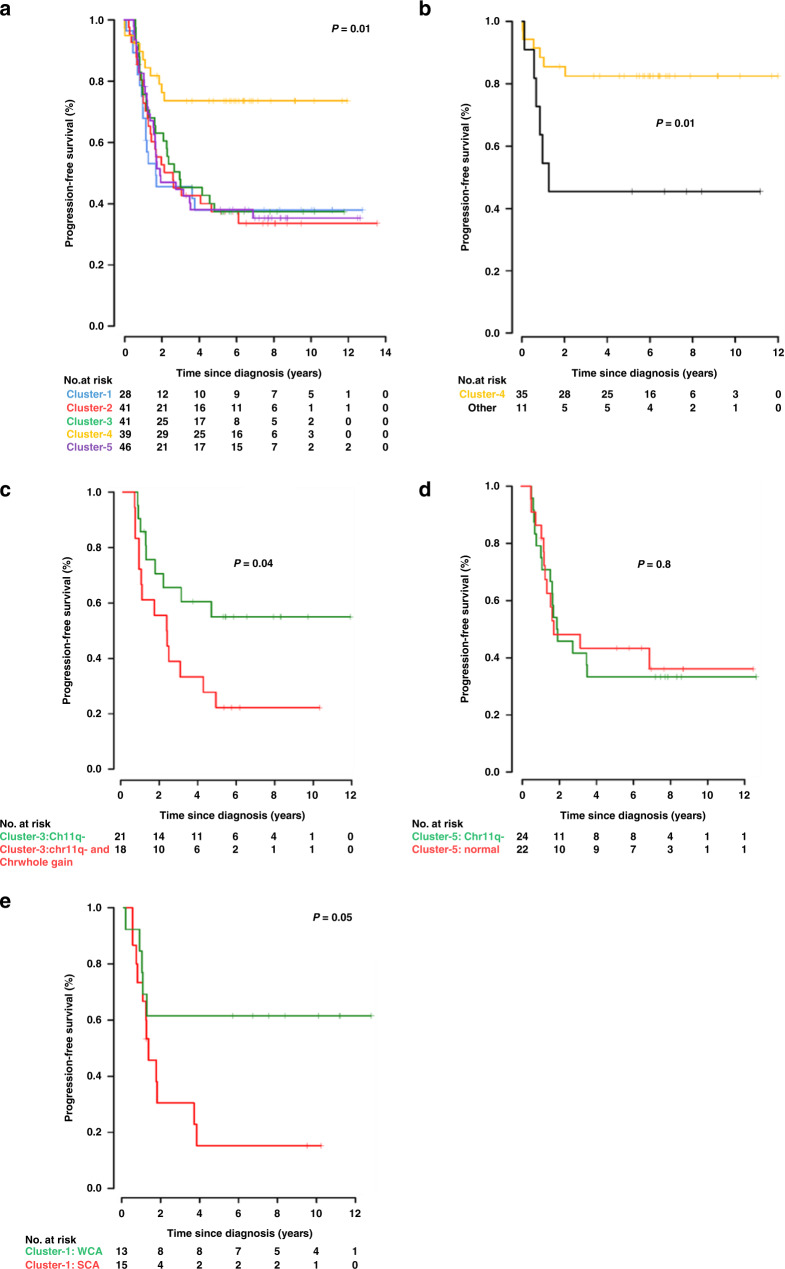
Kaplan–Meier curve showing event-free survival of neuroblastoma patients. **a** Progression-free survival curves for the identified five clusters in neuroblastoma. Cluster-4 (enriched in infant cases) shows event-free survival >80%, while all the other subgroups exhibited poor outcome (42–51%). **b** Infant neuroblastoma shows survival correlated to clustering pattern. Infant cases belonging to any cluster other than Cluster-4 show significantly poorer outcome than those mapping to Cluster-4 (*P* = 0.01). **c** Cluster-3 samples that contain chromosome 11q deletion and whole gain of chromosome 7 have a poorer outcome than those that contain chromosome 11q deletion alone (*P* = 0.05). **d** PFS is not significantly different in Cluster-5 samples stratified for 11qdel status. **e** Cluster 1 samples were stratified into SCA (at least one SCA present, with or without WCA) and WCA (WCA present, but no SCA) groups. Outcome was better in the WCA group (PFS 62%) versus the SCA group (PFS 15%) (*P* = 0.05).

PFS was analysed for the methylation classifiable samples (paired outcome data was available for 195/204 methylation classifiable samples). Cluster-4/infant exhibited significantly higher PFS compared with any of the other clusters (5-year PFS 74%, *P* = 0.01), consistent with the previously reported association between age at diagnosis below 18 months, absence of SCAs and improved survival (Fig. 4a). All the other subgroups (Cluster-1, 2, 3 and 5) exhibited comparably poorer outcomes, with 5-year PFS ranging from 37 to 38% (Fig. 4a). A multivariable Cox regression analysis including other clinical measures found that only COG-risk group was independently predictive of survival (Supplementary Table 2).

A significant number of infant cases (11/46, 24%) did not cluster to the main infant cluster (Cluster-4). As shown in Fig. 4b, outcome in the non-Cluster-4 infant cases was significantly poorer than for infants that map to Cluster-4 [5-year PFS 46% vs 83% respectively, *P* = 0.01]. Furthermore, in Cluster-3/11qdel, 18/42 (43%) of cases exhibited gain of whole gain of chromosome 7, whereas 21/42 (50%) of samples have 11q deletion without chromosome 7 gain. The co-occurrence of 11q deletion and whole gain of chromosome 7 was associated with very poor outcome compared to samples with 11q deletion alone [5-year PFS 22% vs 55%, respectively, *P* = 0.04; Fig. 4c]. Cluster-5 contains 24/47 (51%) patients that have chromosome 11q deletion, while half of the samples in this cluster lack any detected recurring genetic abnormality. However, the presence of 11q deletion was not associated with poorer outcome in cases mapping to Cluster-5 (Fig. 4d). The failure of 11q deletion to be associated with reduced survival in this cluster may be due to the presence of a yet unidentified poor prognostic feature in the Cluster-5 samples lacking 11q deletion. Cluster-1 samples were associated with WCA, with and without additional SCA. We found that the SCA group (*n* = 15) have far poorer survival than Cluster-1 samples with WCA and no SCAs (*n* = 13) (PFS 15% vs 62%, respectively, *P* = 0.05, Fig. 4e) as expected.

#### All five methylation subgroups are validated in an independent unselected cohort

The analysis presented above is focused on high-risk cases where the clinical need is greatest. However, to determine if the same five molecular subgroups could be identified in an unselected cohort the t-SNE analysis was re-run integrating an additional 105 samples from an unselected cohort [14]. In total, 96/105 (91%) samples were methylation classifiable, (i.e., exhibited consistent cluster assignment in >70% of iterative replicates). While the relative distribution of the samples was significantly different (with a higher fraction of Cluster-4/infant diagnosis and, to a lesser extent, Cluster-2/*MYCN* amplification) samples from the unselected cohort nevertheless mapped to all five clusters (Supplementary Fig. 7B). Furthermore, samples mapping to each of the cluster largely replicated the clinical/genetic patterns identified in the original cohort. Due to the high proportion of infant samples in this unselected cohort, there were comparatively few samples in either Cluster-1 (*n* = 6) or Cluster-3 (*n* = 11), preventing a significant expansion of the analysis of outcome in subsets of these clusters reported above. However, the high preponderance of infant samples did allow a significant expansion of the impact on survival of Cluster-4 vs non-cluster-4 infant cases. This further emphasised the poorer outcome for infant cases that did not belong to the main infant cluster (i.e., Cluster-4) (Supplementary Fig. 7D, PFS 49% vs 80% *P* = 0.005). Furthermore, it allowed an expansion of the non-cluster-4 infant samples that lack known high-risk genetic features. Combining the two datasets these samples demonstrated a significant reduction in survival in these cases (PFS 50% vs 80%, *P* = 0.02, Supplementary Fig. 7E). Interestingly, none of these samples were defined as high risk in the COG-risk stratification, suggesting they may represent a novel subset of high-risk samples.

#### Methylation-based subgroups are associated with large numbers of cluster-specific differentially methylated regions (DMRs)

The existence of multiple methylation-based subgroups suggests that differential methylation may lead to biological differences between patients whose tumours map to different clusters. As detailed in Supplementary Tables 3–7, each cluster was associated with a large number of cluster-specific DMRs (*n* = 276, 291, 336, 121 and 518 cluster-specific DMRs in Clusters 1–5, respectively). Gene ontology analysis based on the cluster-specific DMRs (using the missMethyl analysis package) identified links to potentially important functional pathways (e.g., G-protein-coupled signalling, transmembrane receptor signalling, phospholipase-A2 activity and cell–cell adhesion) associated with cluster-specific differential methylation (Supplementary Tables 3–7).

## Discussion

Extensive genome-wide DNA-methylation changes are seen across all types of cancer [30]. Analysis of other cancer types has shown that this can identify molecularly distinct groups with different clinical and genetic features, such as in medulloblastoma, where methylation-based subgrouping is used to inform risk-adapted therapeutic stratification [24]. Initial studies have suggested that neuroblastoma also exhibits two or three different methylation-based clusters [15, 16], although these studies have been limited by sample size and analytical choices. Here, we have used the t-SNE-based approach in a test cohort of over 200 samples. This allowed us to determine that neuroblastoma cases can be robustly distinguished into five DNA-methylation-dependent molecular subgroups. These subgroups all exhibited differential associations with genetic and clinical features of neuroblastoma, providing further support for the subgroups as distinct molecular entities. The initial dataset for this analysis was derived from predominantly high-risk cases, and thus it is possible that other molecular subgroups may be identifiable in unselected or low-risk cohorts.

Three of the molecular subgroups are strongly linked to clinical and genetic features that are known to be prognostic in neuroblastoma [1], with greater than 90% of groups 2, 3 and 4 exhibiting *MYCN* amplification, 11q deletion or infant diagnosis, respectively. However, it must also be noted that not all cases with these abnormalities cluster with these groups. This is particularly common for 11q deletion, where 44% of 11q-deleted cases map to subgroups other than Cluster-3, but is also seen for *MYCN* amplification (17% of cases don’t map to Cluster-2) and infant cases (24% cases don’t map to Cluster-4). This suggests that these features are not always the key defining features of the disease when present and that cases with abnormalities such as 11q deletion may still be molecularly distinct.

In contrast, two of the methylation subgroups appear to represent novel molecular clusters. To determine if a mutational signature could underlie this cluster, we examined mutation frequency across the five clusters and found this to be broadly similar between the methylation groups. There was limited evidence that *ALK* mutation, the most frequently mutated gene in neuroblastoma [31], may be associated with Cluster-1, although this was of borderline significance. Also, while *ALK* mutations were more frequent, they were still present in a minority of cases in Cluster-1. However, it could suggest a potential link to aberrant regulation of *ALK*-related pathways and future studies could assess if this was a more general feature of cases mapping to this cluster.

Cluster-5 lacked a single clear defining feature, but unlike Cluster-1, it did show significant enrichment for 11q deletion (24/47, 51%). Interestingly, 11q-deleted cases in this subgroup were far less likely to be associated with other chromosomal aneuploidies, such as 3p and 4p-deletions or with gain of 17q, than cases that mapped to the main 11q-deleted cluster (Cluster-3/11qdel). Furthermore, the depth of the reduction in signal at 11q was generally less. This suggests that 11q-deletions occurring in this subgroup may be arising later in tumour evolution. Overall, the initial molecular change that underlies this subgroup remains to be elucidated.

Consistent with previous reports, survival analysis in this study demonstrated significantly better outcome for Cluster-4 (associated with infant diagnosis) and poorer outcome in all other clusters. However, around a quarter of infant-diagnosed cases mapped to clusters associated with poor outcome as opposed to the main infant cluster (i.e., Cluster-4). Analysis of survival specifically in the infant cases determined that cases that mapped to the other subgroups exhibit the poor outcome associated with those clusters, suggesting this could be used to identify high-risk infant cases. The total number of samples in this category was small (*n* = 11) making it difficult to determine whether the methylation subgroup or other high-risk features (such as *MYCN* amplification [32], which was present in two cases) would be optimal for the identification of high-risk infant cases. However, excluding cases with known high-risk genetic features (*MYCN* amplification or 11q deletion) did not alter the poor survival of this group (Supplementary Fig. 7E). If the poor outcome of the non-clustered infant samples can be confirmed in a larger replication cohort, this would have potential clinical utility. However, clinical use of such profiling would require the development of a robust methylation classification tool that could be widely used, preferably without the need for expert knowledge. This could be modelled on the methylation classifier recently developed for use in CNS tumours [33].

In addition, stratification of two of the other clusters based on additional genetic features may allow the identification of two groups of very high-risk patients with PFS of approximately 20% (i.e., Cluster-1 samples that are SCA positive and cluster-3/11qdel samples that have a co-occurring whole chromosome 7 gain). However, the sample sizes in all the above cases in this study are small, and the validation cohort was too small to confirm the poor outcome of these patient groups. Thus, confirmatory studies will be required to confirm the potential clinical significance of these groups for the prognosis of neuroblastoma patients. Approximately half of Cluster-5 samples exhibit an 11q deletion. Although this is generally regarded as a high-risk feature, there was no significant difference in outcome dependent on the presence of 11q deletion in this cluster. This could be related to the presence of other high-risk factors, such as *MYCN* amplification. However, *MYCN* amplification was rare in this subgroup (*n* = 3), consistent with the known reciprocal association of these high-risk markers and thus removing these cases did not impact the comparative survival of Cluster-5 cases with or without 11q deletion (data not shown).

It is not clear why the gain of chromosome 7, in addition to 11q deletion, would be associated with such a poor outcome. One possibility would be the overexpression of a key oncogene located on chromosome 7. Recently the *GPC2* gene, which maps to chr7q, has been identified as oncogenic in neuroblastoma, and it has been suggested that the gain of chromosome 7 could be associated with *GPC2* overexpression [34]. However, *GPC2* was not among the 32 genes (10 mapping to chromosome 7, of which 9 were upregulated) found to exhibit differential expression in Cluster-3 cases with/without chromosome 7 gain (data not shown). However, the upregulated genes on chromosome 7 did include the *RAC1* (Rac family small GTPase-1) gene, which has known roles in promoting tumour development and metastasis [35], and thus would merit investigation for potential involvement in the poor outcome of this group of patients.

The four non-infant clusters are all associated with clearly different genetic profiles. Two of the clusters (Cluster-2/*MYCN* and Cluster-3/11qdel) were strongly associated with genetic abnormalities already strongly linked to neuroblastoma development. However, Cluster-1 and Cluster-5 lack strong associations with previously identified or hypothesised genetic drivers of neuroblastoma. Identifying these subgroups is a key first step in delineating the cancer pathways underpinning these molecular subgroups, which will be essential to allow the subsequent development of rational targeted therapies to improve the poor outcome evident in these two novel clusters. In addition, a small fraction of the samples (8.5%) could not be reproducibly mapped to a specific methylation subgroup. It is not clear if these samples represent a separate subgroup of samples (that lack any apparent consistent methylation similarities) or if this relates to technical limitations, (e.g., samples with low tumour cell content). For samples with low tumour cell content, it is possible they could be classified using deconvolution approaches [36] or through identifying highly subgroup-specific methylation markers. Alternatively, the currently defined clusters could be used to identify novel molecular targets and then subsequent clinical development could be based on the presence/absence of the specific molecular target as opposed to cluster designation.

The identification of molecular subgroups within cancers can allow the identification of important biological differences between individual subgroups, which could allow the identification of novel therapeutic approaches. To begin to address this possibility, we identified methylation differences (DMRs) specific for each of the five methylation clusters. All clusters were associated with a relatively large number of specific DMRs and gene ontology analysis identified potential dysregulation of specific pathways associated with each cluster. Of particular interest was the very strong association between Cluster-5 and G-protein-coupled signalling. G-protein-coupled signalling has been extensively studied in multiple types of cancer [37] and is an active area for the development of novel therapeutic approaches [38]. This suggests that future studies could assess the potential role of dysregulated G-protein-coupled signalling in the Cluster-5, which is one of the two clusters that is not defined by a known clinical/genetic feature.

In conclusion, clustering using genome-wide DNA-methylation data was able to identify five molecular subgroups, with clearly different genetic and clinical characteristics, which can be used to further investigate the molecular basis of this often fatal childhood cancer. Survival analysis determined that, as expected, survival was significantly better in the cluster associated with infant diagnosis (Cluster-4) but was further able to show that infant samples that failed to map to Cluster-4 exhibited the poor survival associated with the other clusters and may represent a novel subset of high-risk infant cases.

## Supplementary information

Supplementary Figures

Confirmation of approval of author list

Supplementary Table 1

Supplementary Table 2

Supplementary Table 3

Supplementary Table 4

Supplementary Table 5

Supplementary Table 6

Supplementary Table 7

aj Checklist

## Data Availability

All datasets used in this study are publicly available as part of the TARGET programme (https://ocg.cancer.gov/programs/target/data-matrix) or on the NCBI GEO website (GSE73518).
