# Supplementary material for: Genome wide DNA methylation analysis identifies novel molecular subgroups and predicts survival in neuroblastoma
Source: Br J Cancer. 2022 Sep 29;127(11):2006–15. doi: 10.1038/s41416-022-01988-z (PMC9681858; doi:10.1038/s41416-022-01988-z)
Supplement: Supplementary file 1 — Supplementary Figures [file 41416_2022_1988_MOESM1_ESM.pptx]

## Slide 1
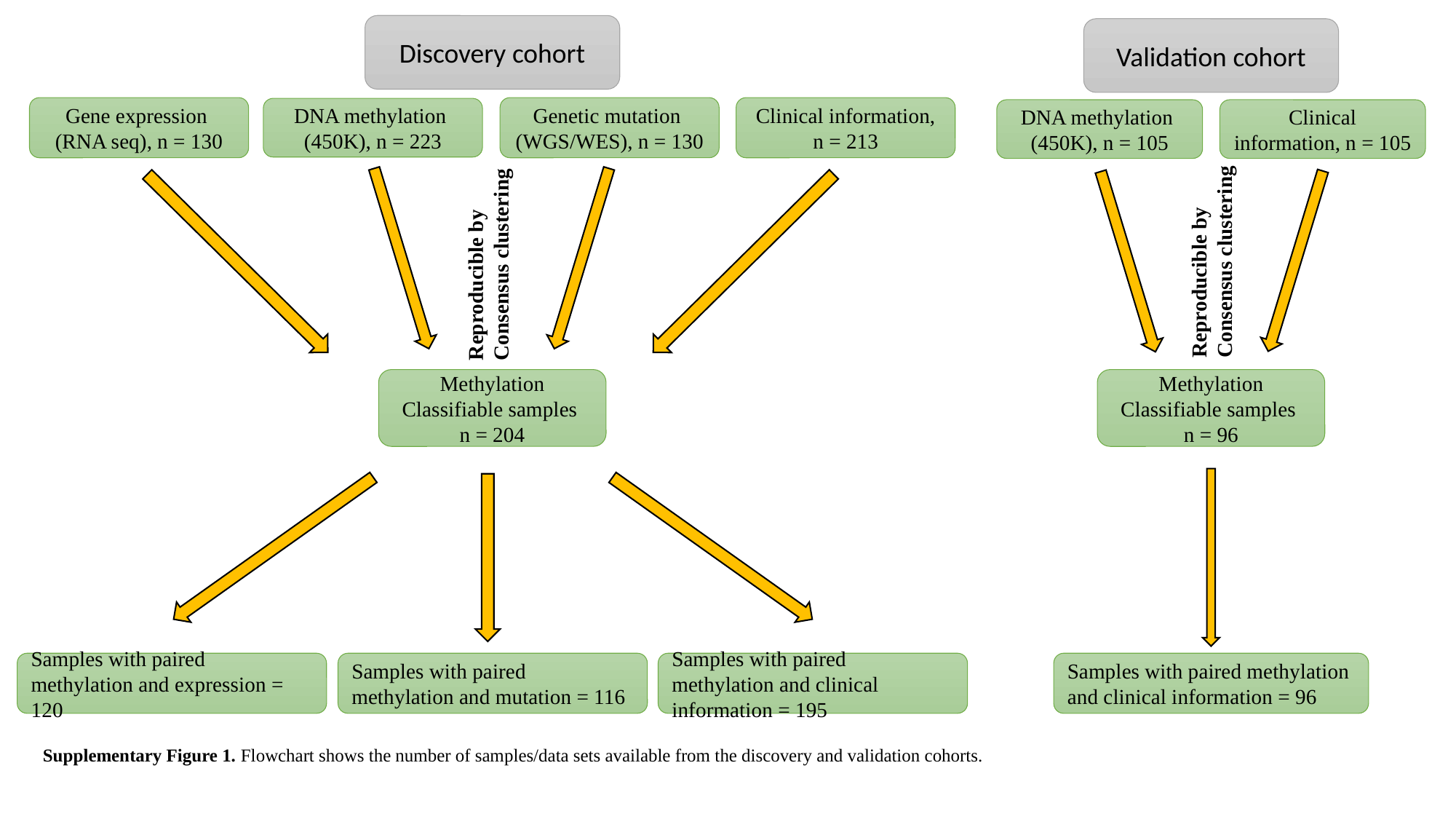

Discovery cohort
Validation cohort
Genetic mutation
(WGS/WES), n = 130
Clinical information, n = 213
Gene expression
(RNA seq), n = 130
DNA methylation
(450K), n = 223
DNA methylation
(450K), n = 105
Clinical information, n = 105
Reproducible by
Consensus clustering
Reproducible by
Consensus clustering
Methylation Classifiable samples
n = 204
Methylation Classifiable samples
n = 96
Samples with paired methylation and expression = 120
Samples with paired methylation and mutation = 116
Samples with paired methylation and clinical information = 195
Samples with paired methylation and clinical information = 96
Supplementary Figure 1. Flowchart shows the number of samples/data sets available from the discovery and validation cohorts.

## Slide 2
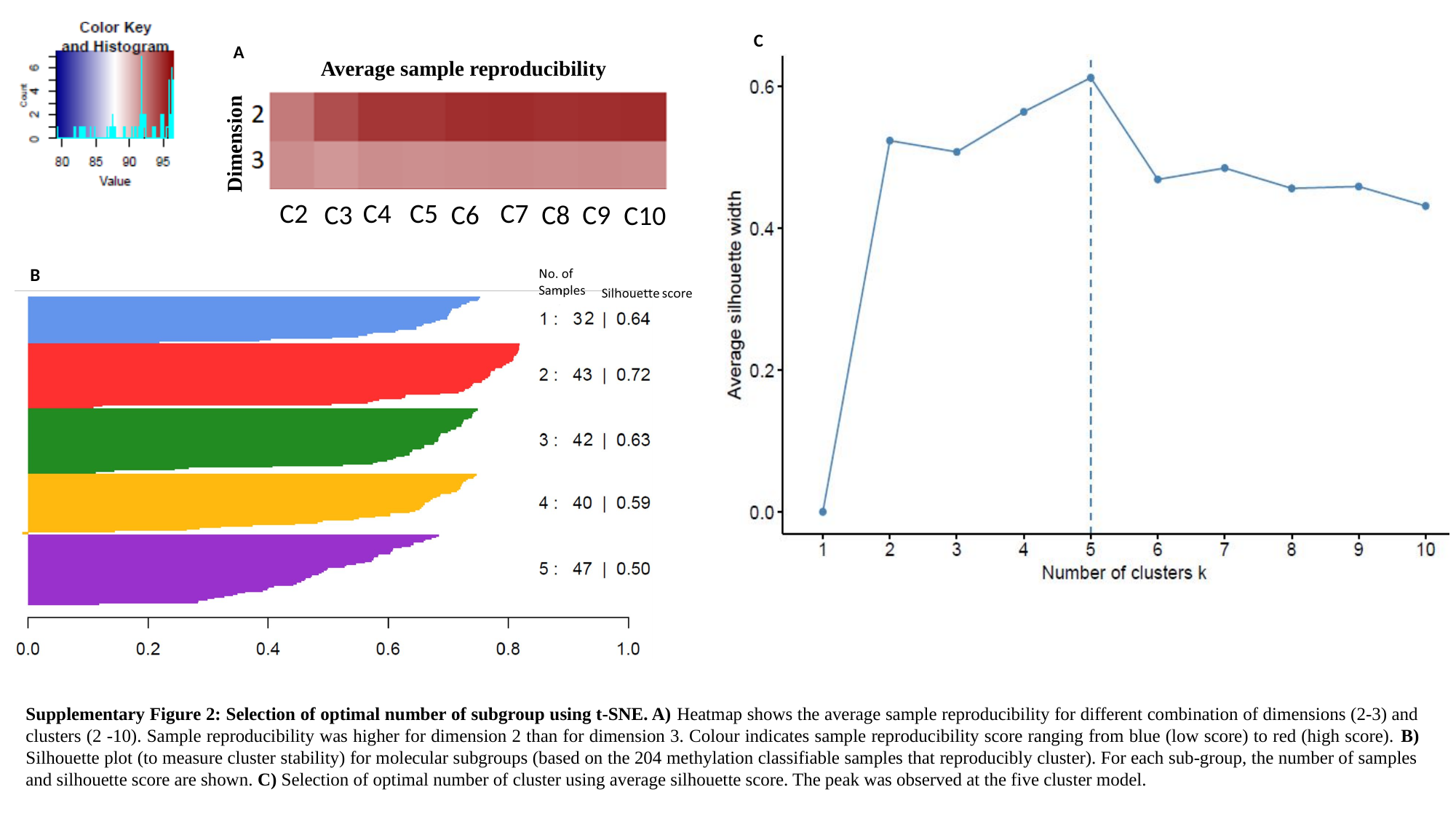

C
A
Average sample reproducibility
Dimension
C2
C4
C5
C7
C3
C8
C9
C6
C10
B
Supplementary Figure 2: Selection of optimal number of subgroup using t-SNE. A) Heatmap shows the average sample reproducibility for different combination of dimensions (2-3) and clusters (2 -10). Sample reproducibility was higher for dimension 2 than for dimension 3. Colour indicates sample reproducibility score ranging from blue (low score) to red (high score). B) Silhouette plot (to measure cluster stability) for molecular subgroups (based on the 204 methylation classifiable samples that reproducibly cluster). For each sub-group, the number of samples and silhouette score are shown. C) Selection of optimal number of cluster using average silhouette score. The peak was observed at the five cluster model.

## Slide 3
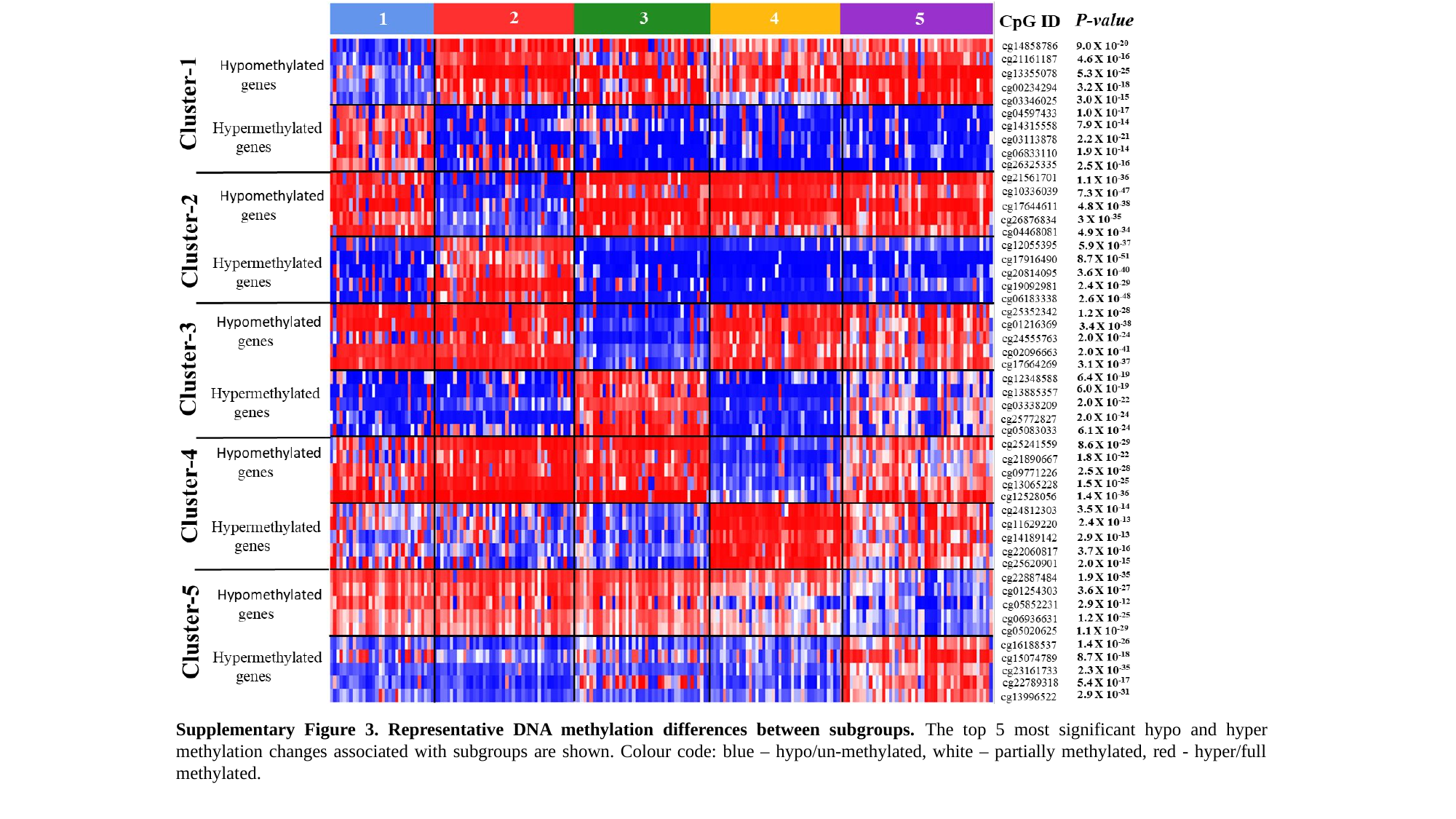

Supplementary Figure 3. Representative DNA methylation differences between subgroups. The top 5 most significant hypo and hyper methylation changes associated with subgroups are shown. Colour code: blue – hypo/un-methylated, white – partially methylated, red - hyper/full methylated.

## Slide 4
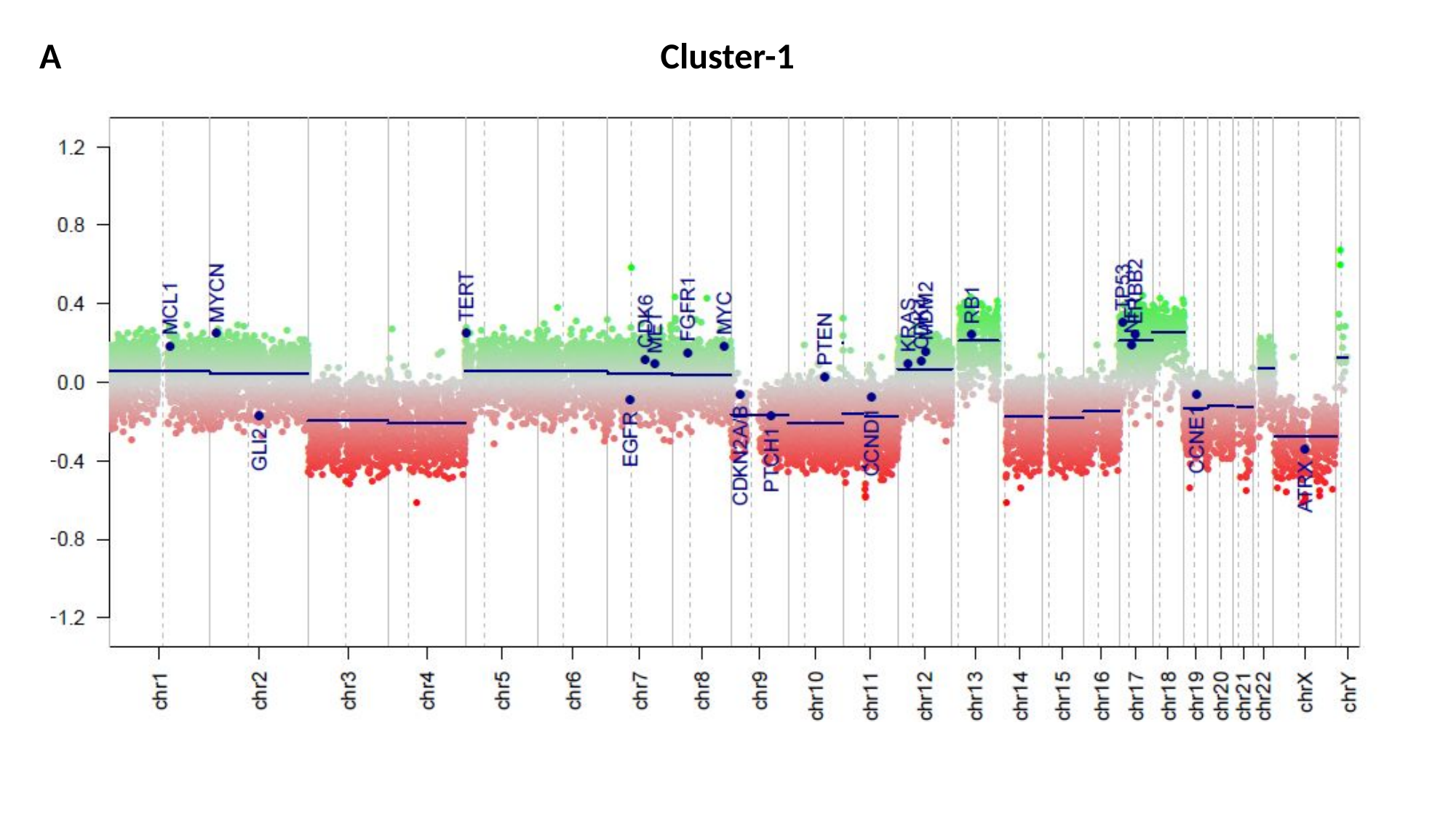

A
Cluster-1

## Slide 5
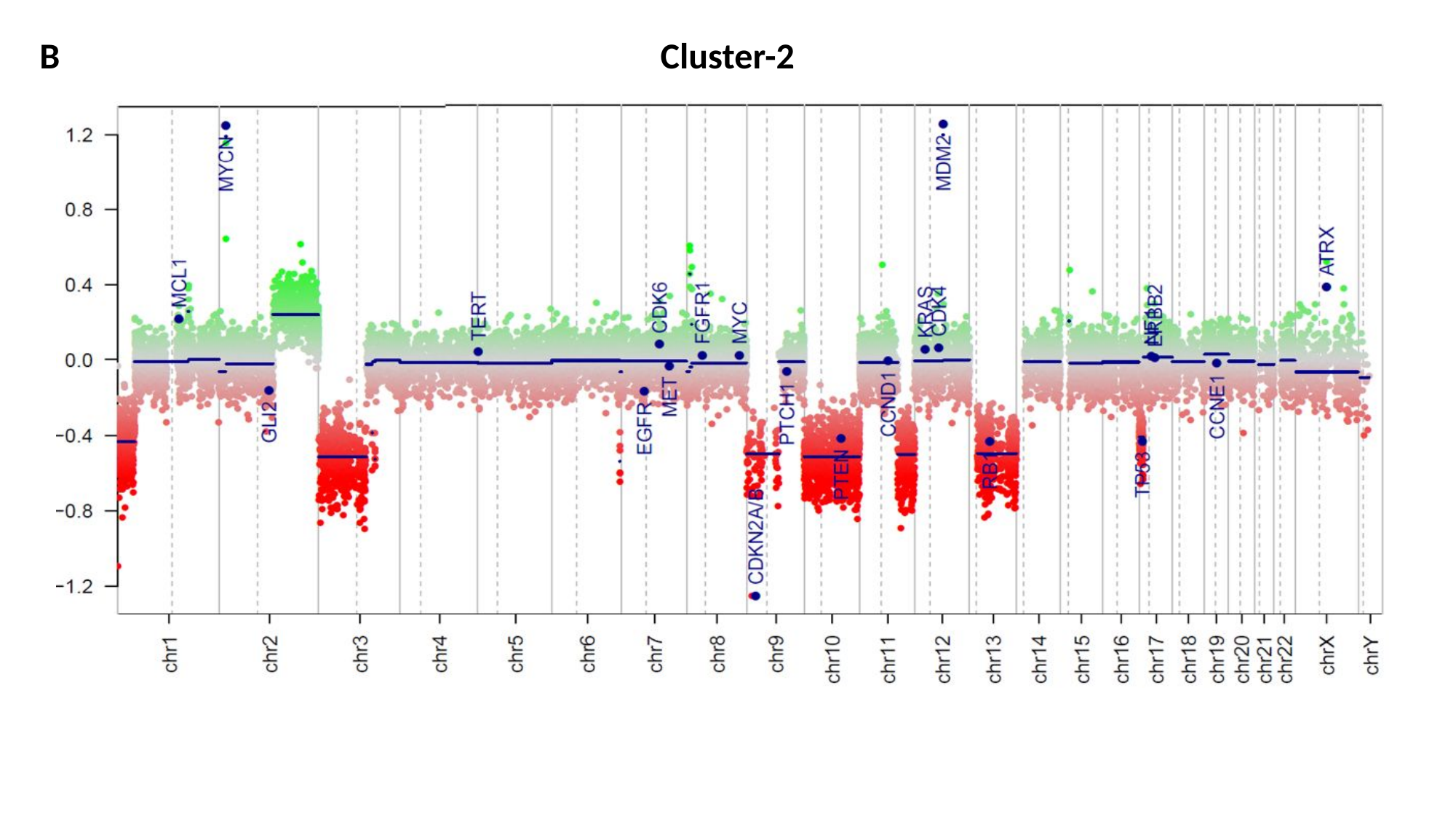

B
Cluster-2

## Slide 6
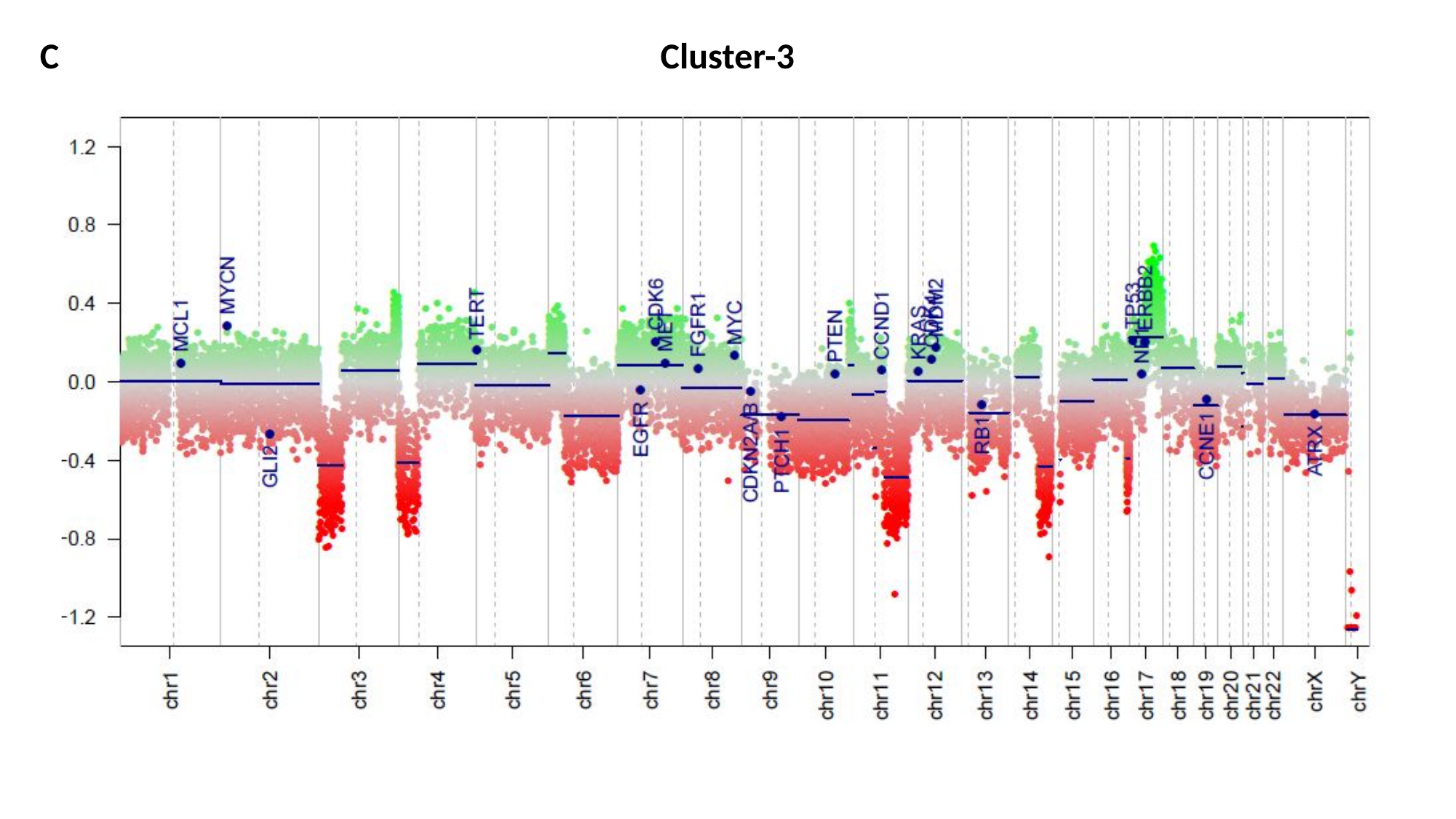

C
Cluster-3

## Slide 7
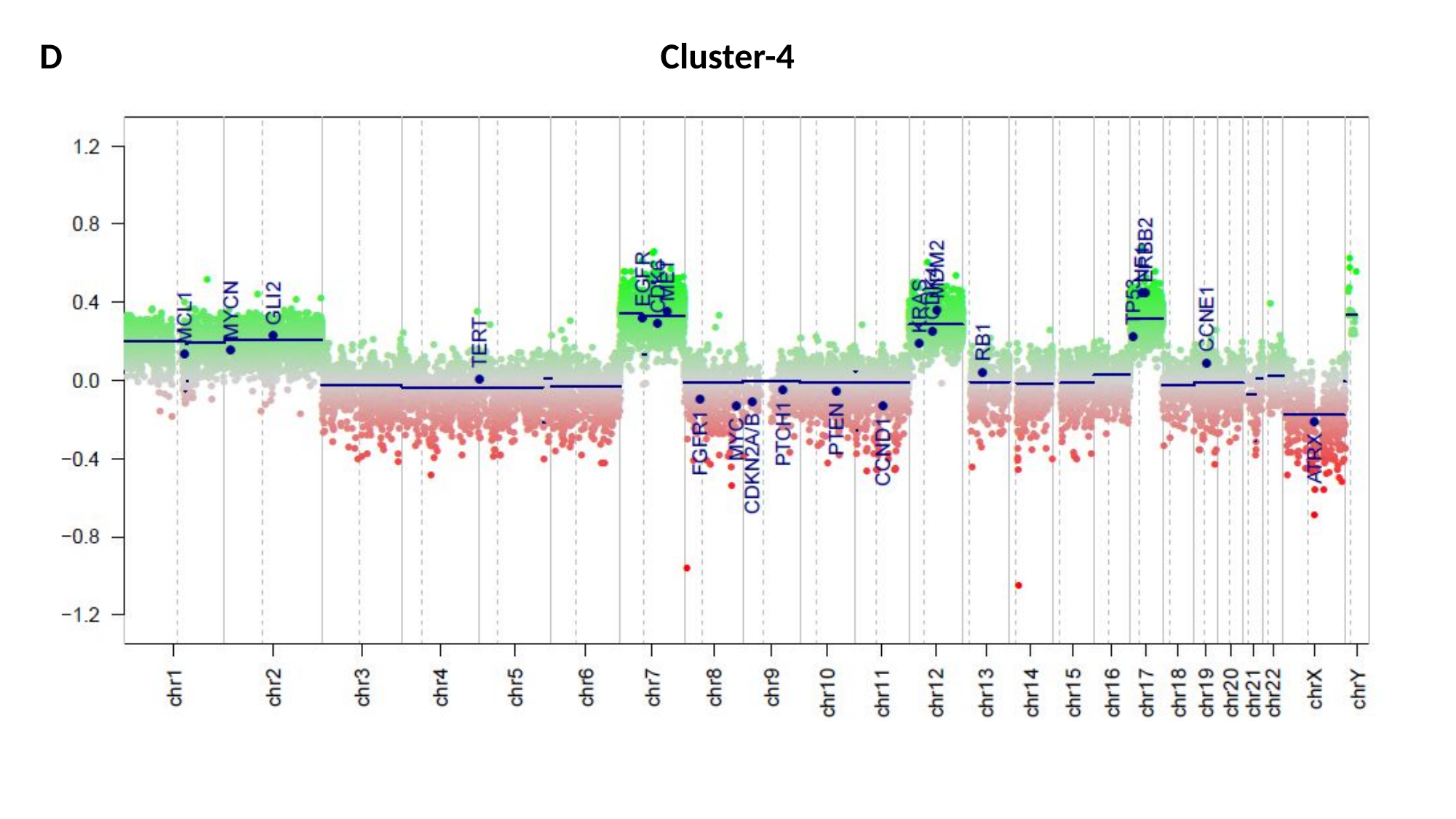

D
Cluster-4

## Slide 8
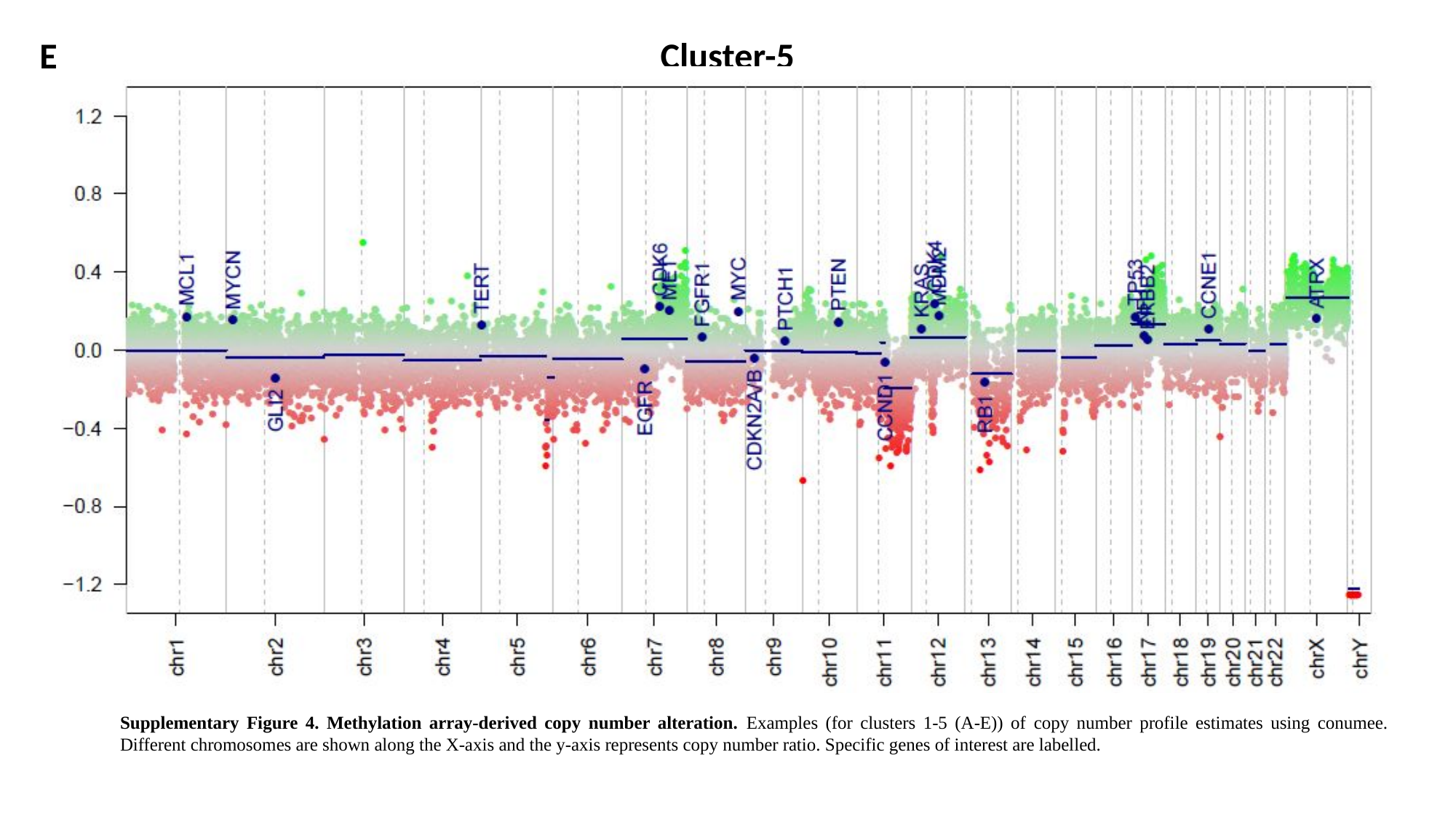

E
Cluster-5
Supplementary Figure 4. Methylation array-derived copy number alteration. Examples (for clusters 1-5 (A-E)) of copy number profile estimates using conumee. Different chromosomes are shown along the X-axis and the y-axis represents copy number ratio. Specific genes of interest are labelled.

## Slide 9
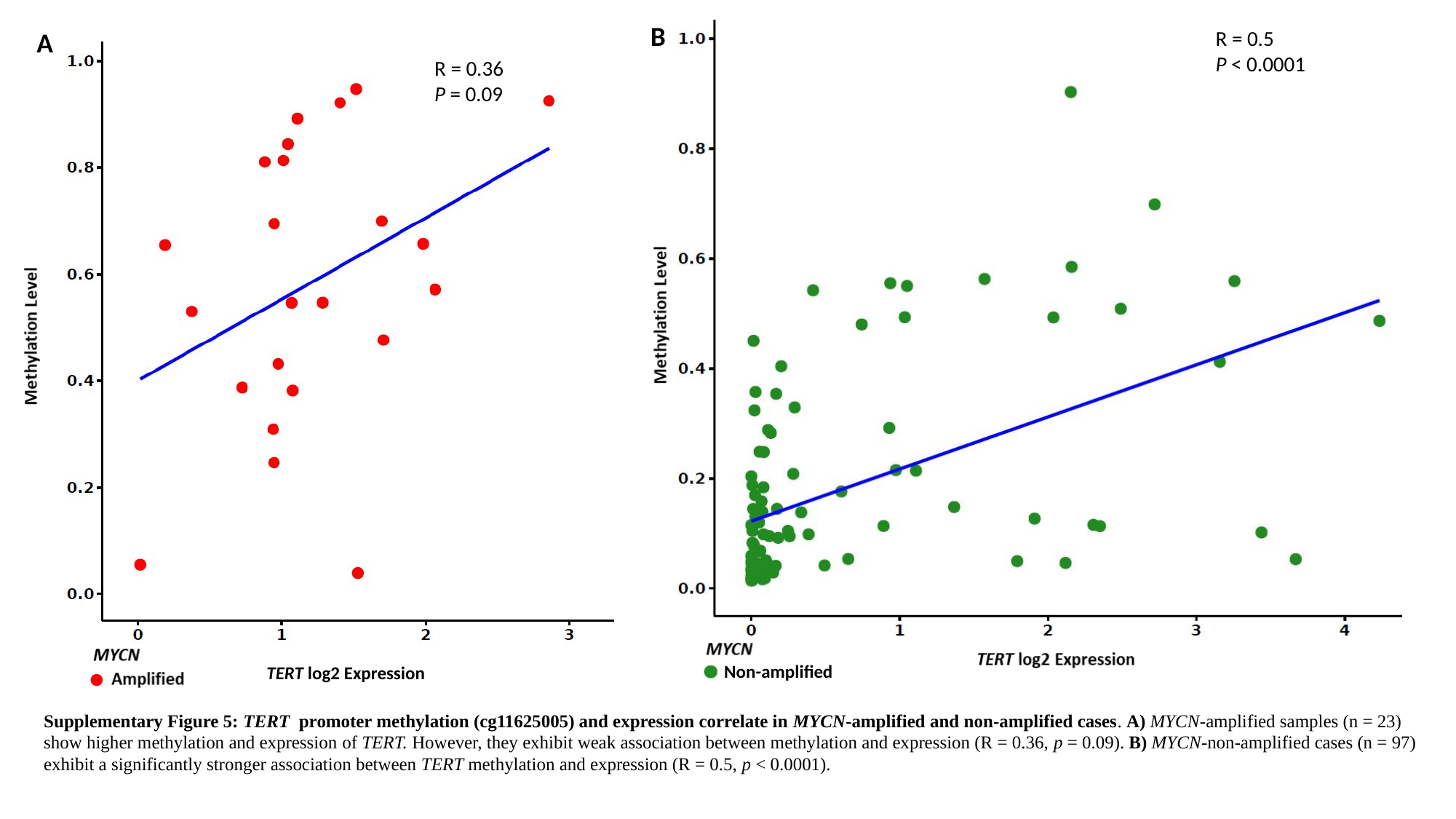

B
R = 0.5
P < 0.0001
Non-amplified
A
R = 0.36
P = 0.09
TERT log2 Expression
Supplementary Figure 5: TERT promoter methylation (cg11625005) and expression correlate in MYCN-amplified and non-amplified cases. A) MYCN-amplified samples (n = 23) show higher methylation and expression of TERT. However, they exhibit weak association between methylation and expression (R = 0.36, p = 0.09). B) MYCN-non-amplified cases (n = 97) exhibit a significantly stronger association between TERT methylation and expression (R = 0.5, p < 0.0001).

## Slide 10
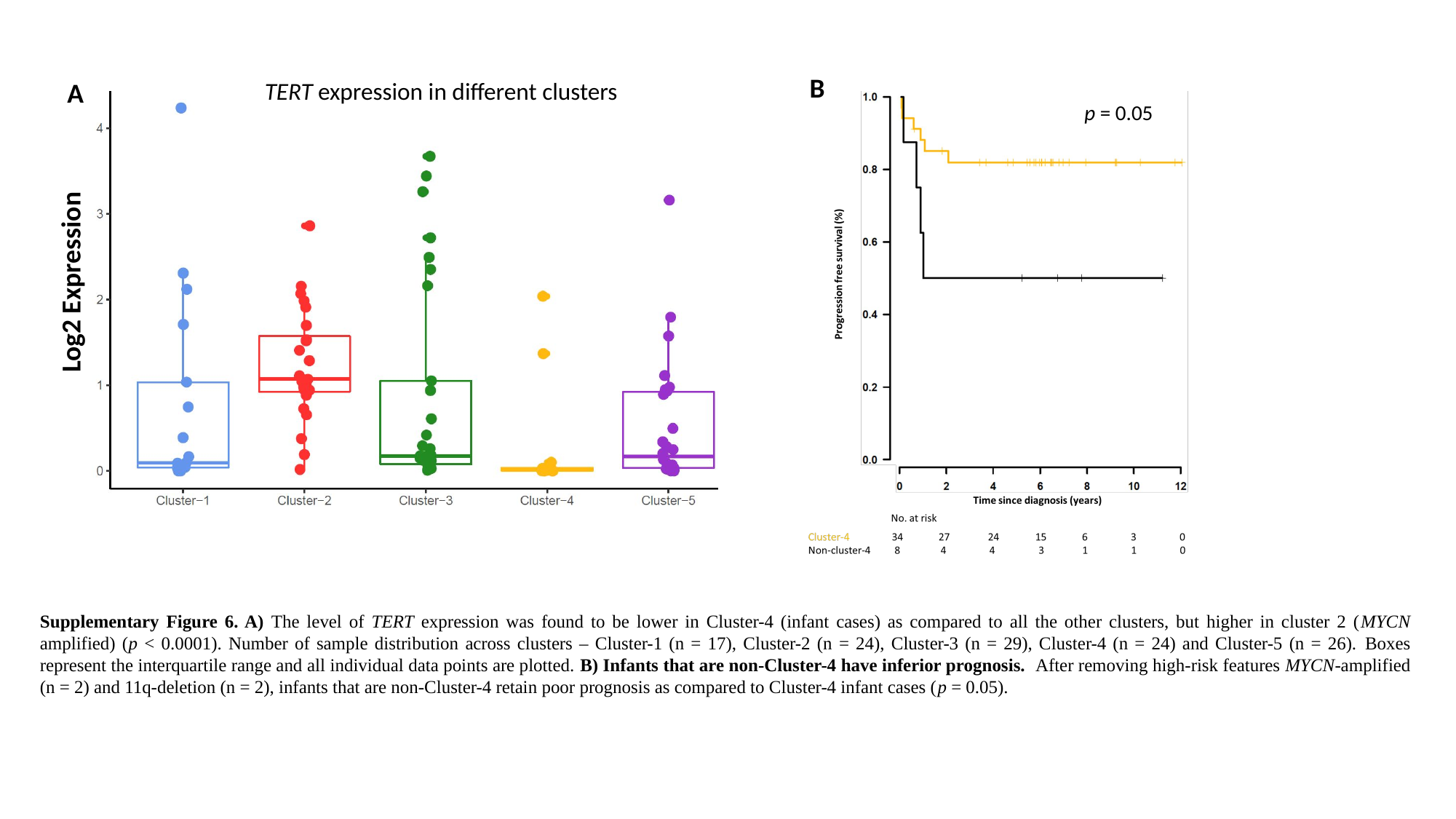

B
A
TERT expression in different clusters
 p = 0.05
Log2 Expression
Supplementary Figure 6. A) The level of TERT expression was found to be lower in Cluster-4 (infant cases) as compared to all the other clusters, but higher in cluster 2 (MYCN amplified) (p < 0.0001). Number of sample distribution across clusters – Cluster-1 (n = 17), Cluster-2 (n = 24), Cluster-3 (n = 29), Cluster-4 (n = 24) and Cluster-5 (n = 26). Boxes represent the interquartile range and all individual data points are plotted. B) Infants that are non-Cluster-4 have inferior prognosis. After removing high-risk features MYCN-amplified (n = 2) and 11q-deletion (n = 2), infants that are non-Cluster-4 retain poor prognosis as compared to Cluster-4 infant cases (p = 0.05).

## Slide 11
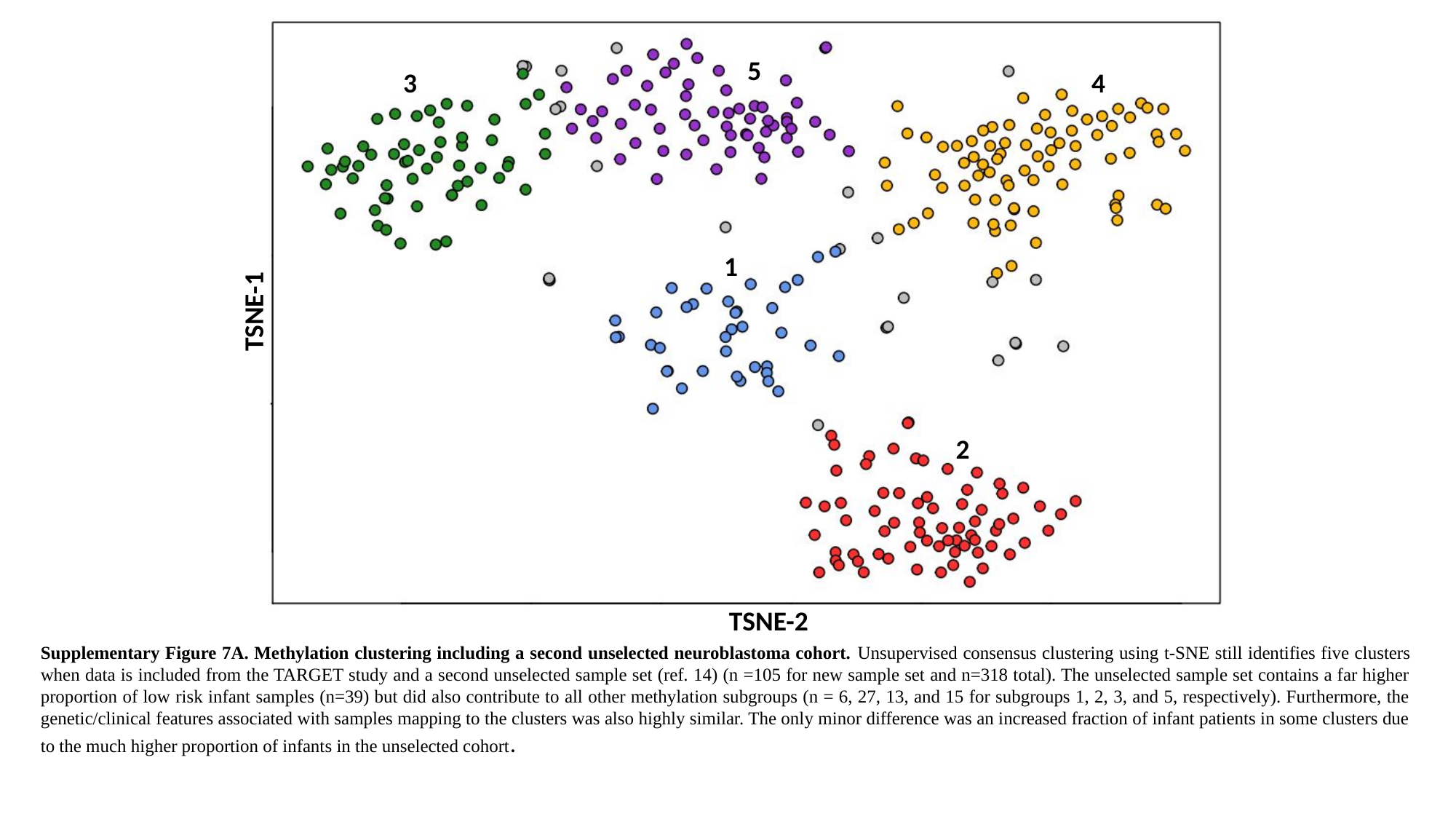

TSNE-1
TSNE-2
1
2
3
4
5
Supplementary Figure 7A. Methylation clustering including a second unselected neuroblastoma cohort. Unsupervised consensus clustering using t-SNE still identifies five clusters when data is included from the TARGET study and a second unselected sample set (ref. 14) (n =105 for new sample set and n=318 total). The unselected sample set contains a far higher proportion of low risk infant samples (n=39) but did also contribute to all other methylation subgroups (n = 6, 27, 13, and 15 for subgroups 1, 2, 3, and 5, respectively). Furthermore, the genetic/clinical features associated with samples mapping to the clusters was also highly similar. The only minor difference was an increased fraction of infant patients in some clusters due to the much higher proportion of infants in the unselected cohort.

## Slide 12
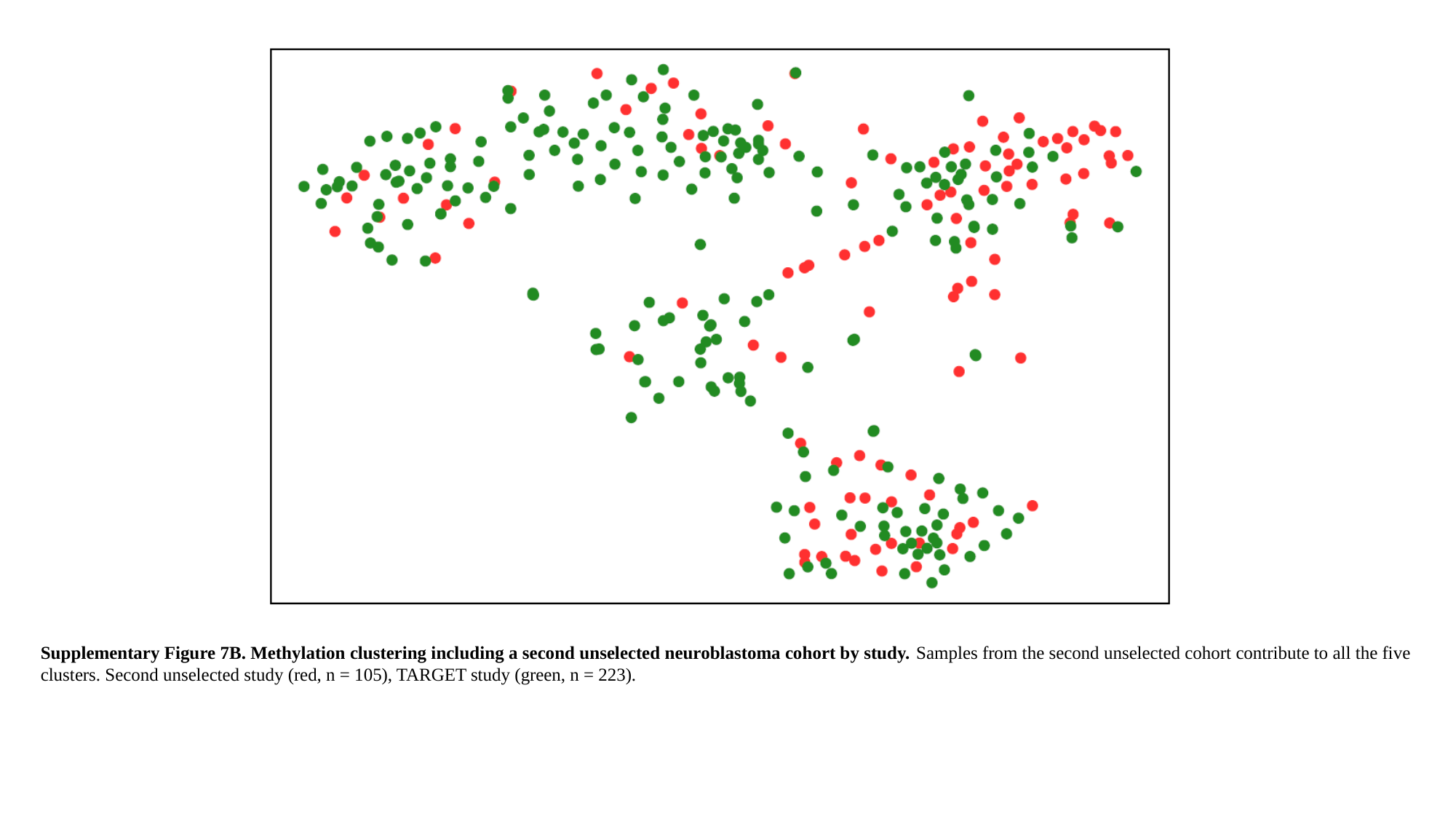

Supplementary Figure 7B. Methylation clustering including a second unselected neuroblastoma cohort by study. Samples from the second unselected cohort contribute to all the five clusters. Second unselected study (red, n = 105), TARGET study (green, n = 223).

## Slide 13
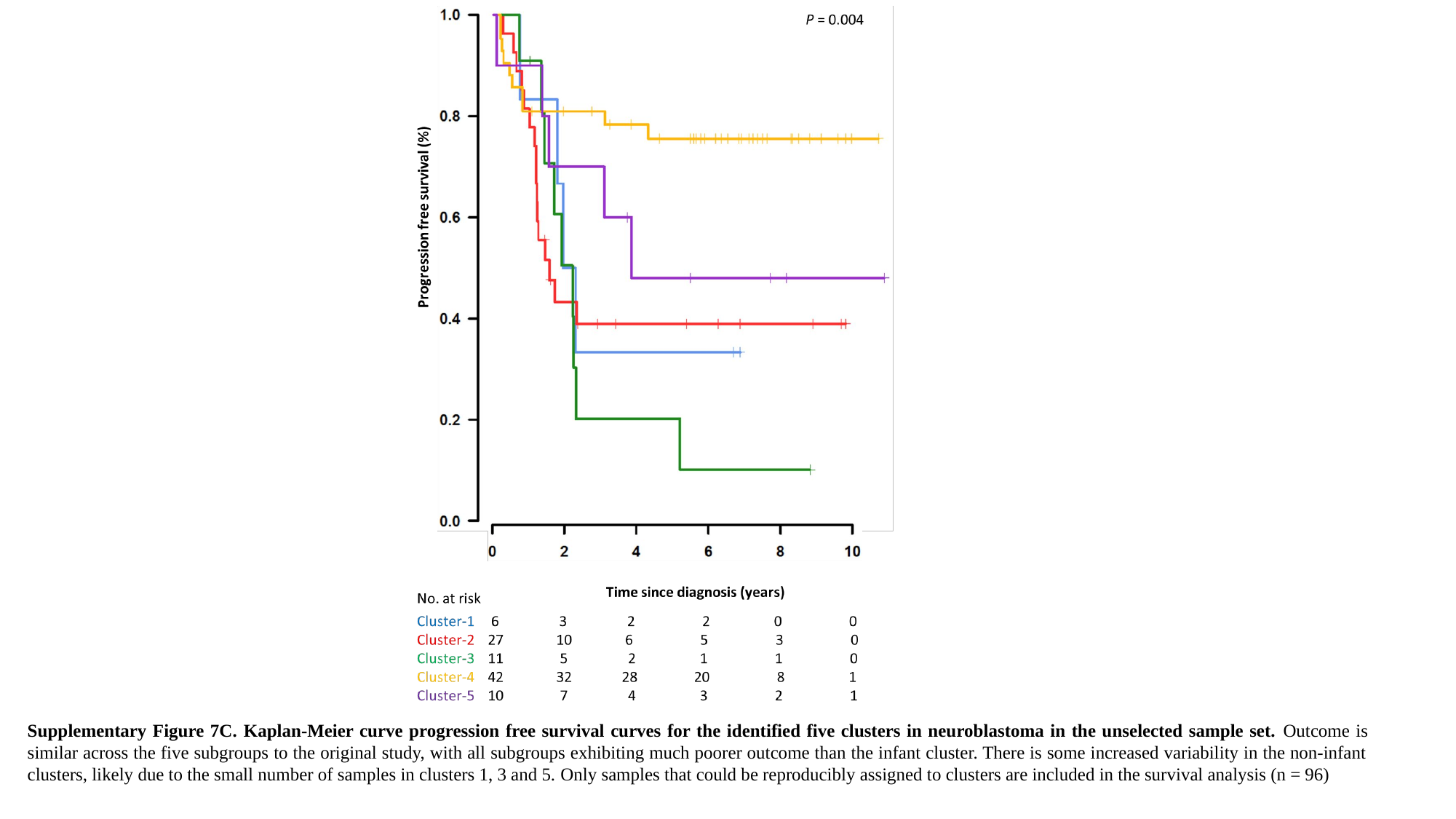

Supplementary Figure 7C. Kaplan-Meier curve progression free survival curves for the identified five clusters in neuroblastoma in the unselected sample set. Outcome is similar across the five subgroups to the original study, with all subgroups exhibiting much poorer outcome than the infant cluster. There is some increased variability in the non-infant clusters, likely due to the small number of samples in clusters 1, 3 and 5. Only samples that could be reproducibly assigned to clusters are included in the survival analysis (n = 96)

## Slide 14
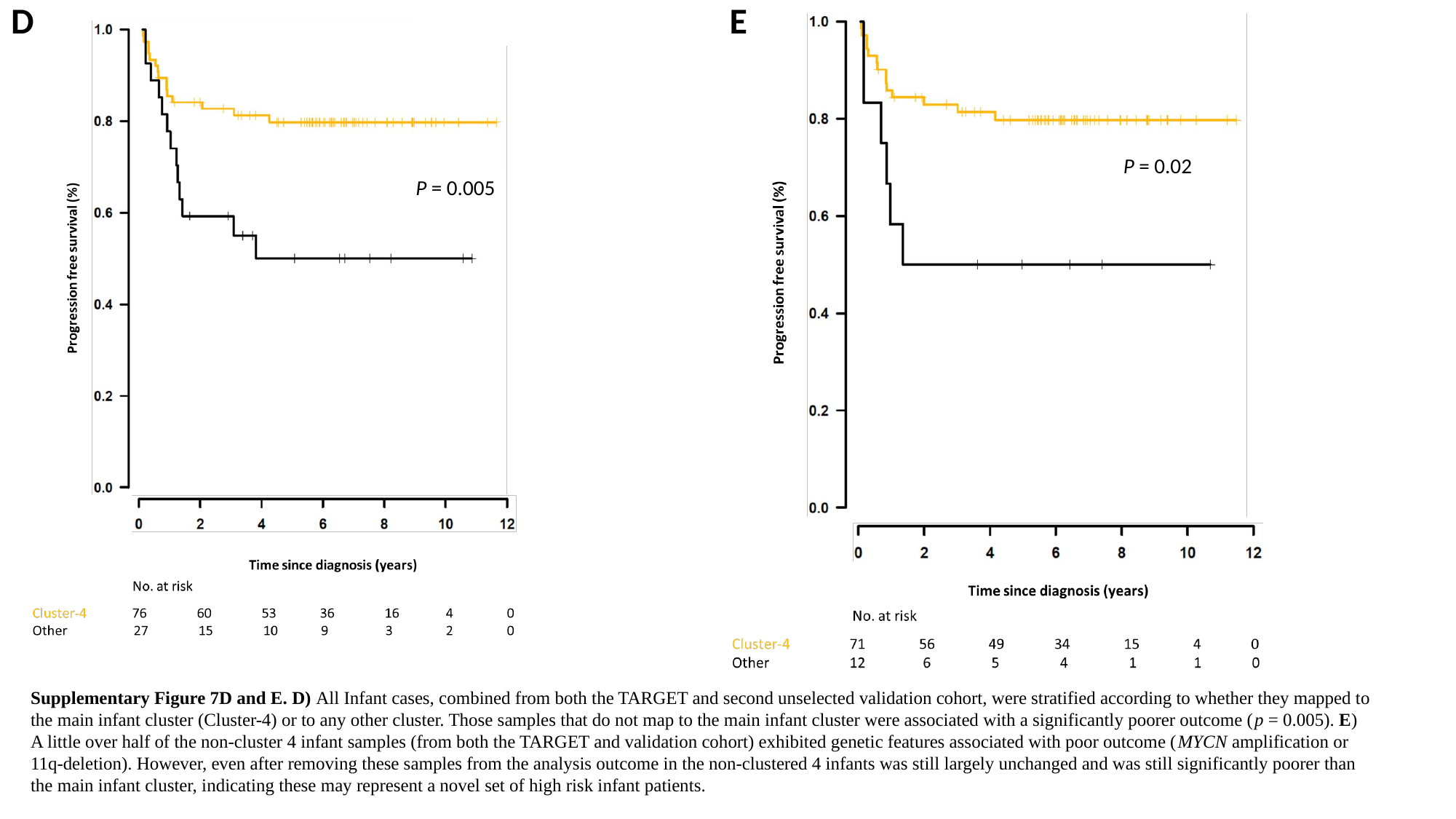

D
E
P = 0.02
P = 0.005
Supplementary Figure 7D and E. D) All Infant cases, combined from both the TARGET and second unselected validation cohort, were stratified according to whether they mapped to the main infant cluster (Cluster-4) or to any other cluster. Those samples that do not map to the main infant cluster were associated with a significantly poorer outcome (p = 0.005). E) A little over half of the non-cluster 4 infant samples (from both the TARGET and validation cohort) exhibited genetic features associated with poor outcome (MYCN amplification or 11q-deletion). However, even after removing these samples from the analysis outcome in the non-clustered 4 infants was still largely unchanged and was still significantly poorer than the main infant cluster, indicating these may represent a novel set of high risk infant patients.
