# Supplementary material for: Genome wide DNA methylation analysis identifies novel molecular subgroups and predicts survival in neuroblastoma
Source: Br J Cancer. 2022 Sep 29;127(11):2006–15. doi: 10.1038/s41416-022-01988-z (PMC9681858; doi:10.1038/s41416-022-01988-z)

**e-mail from Wen Hao**


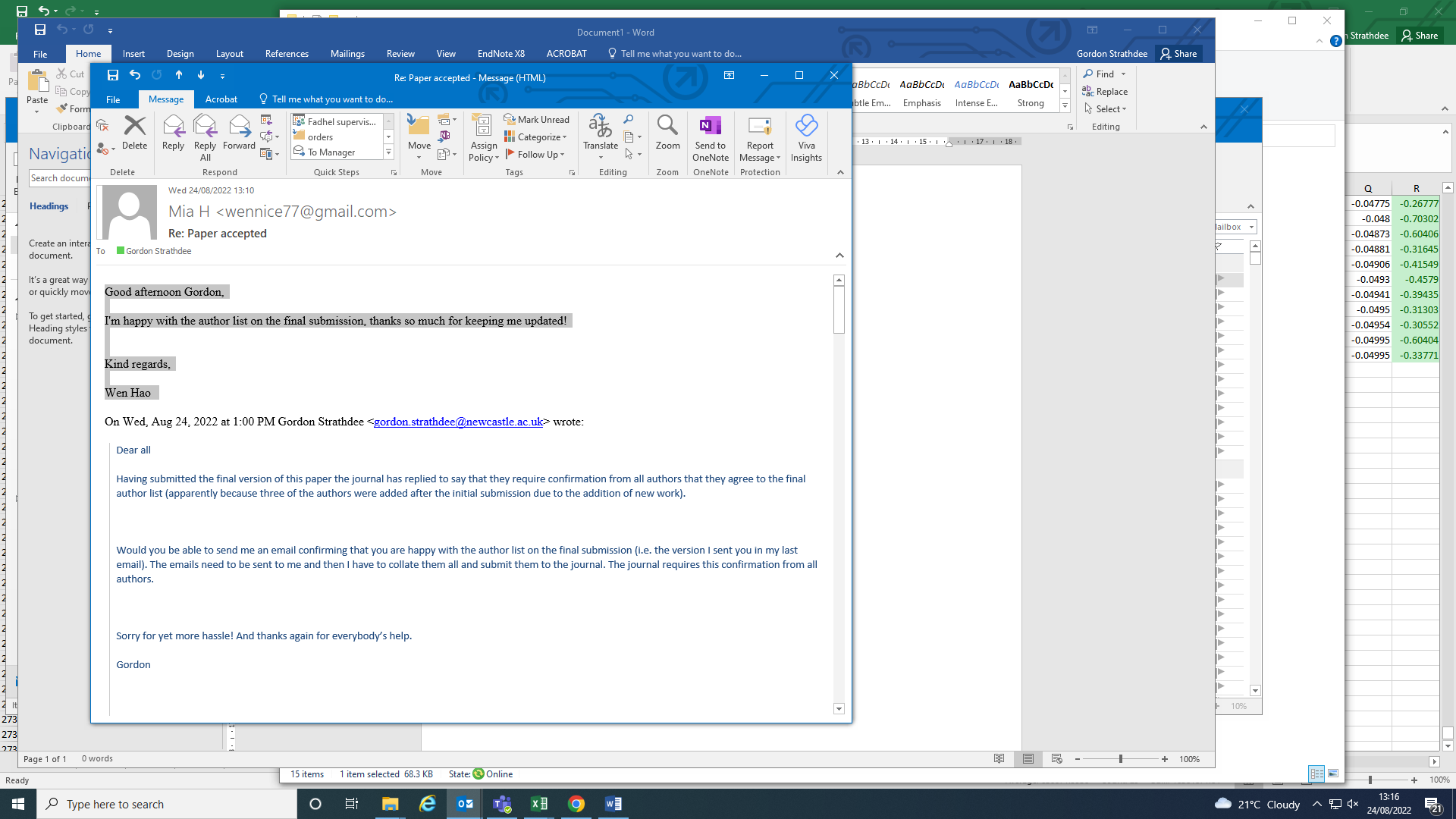


email from John Maris


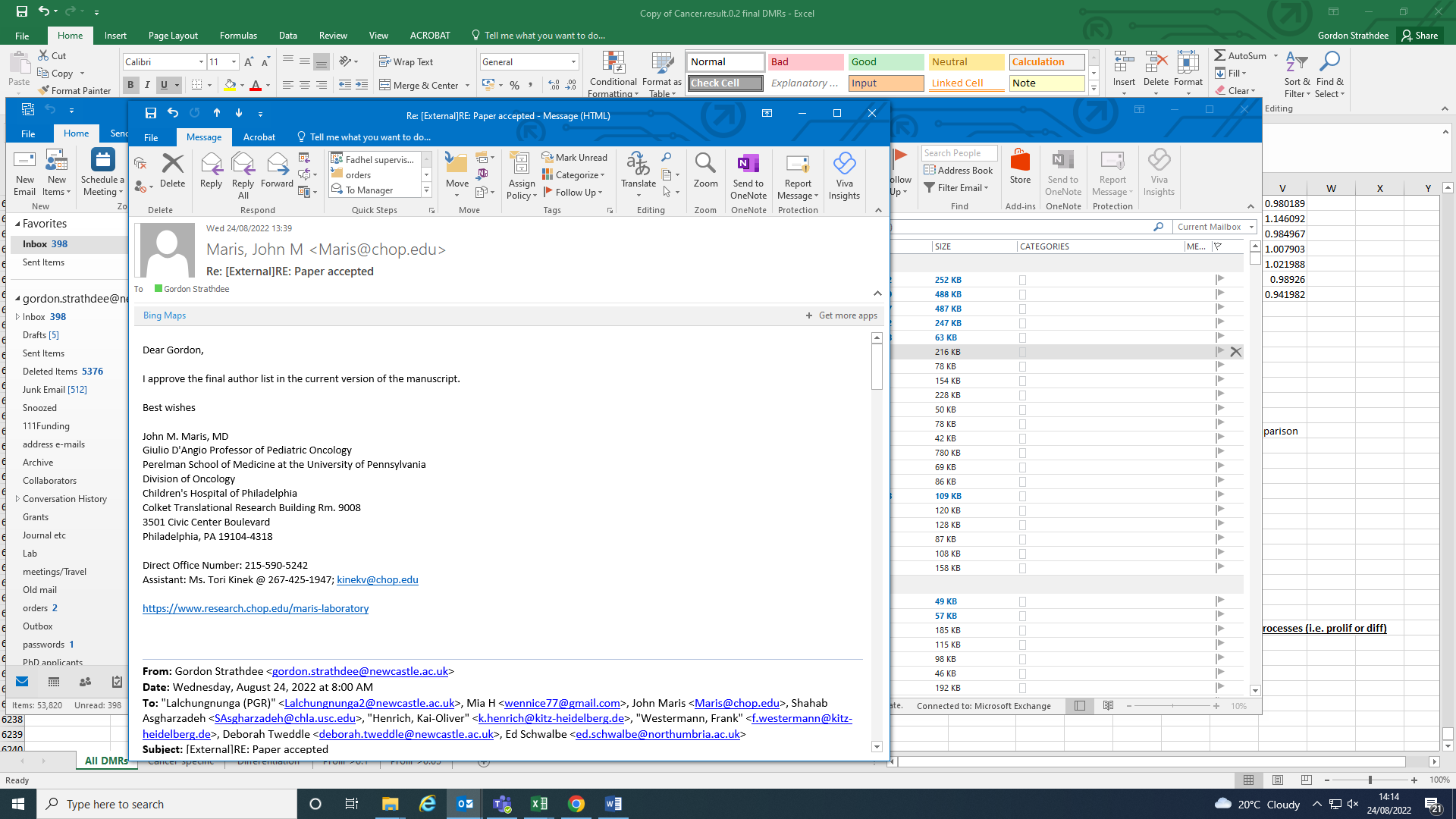


Email from Edward Schwalbe


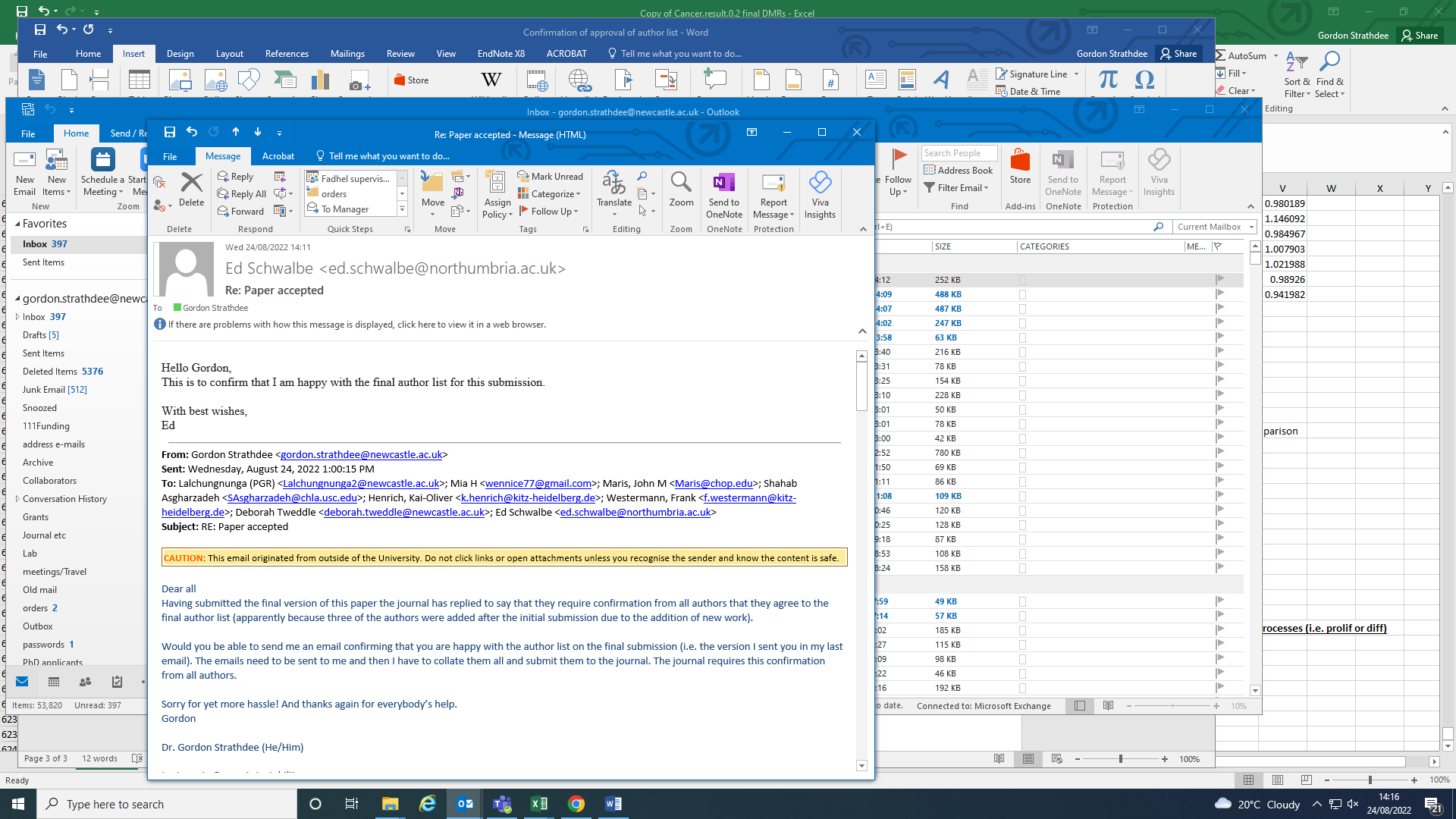


Email from Lalchungnunga:


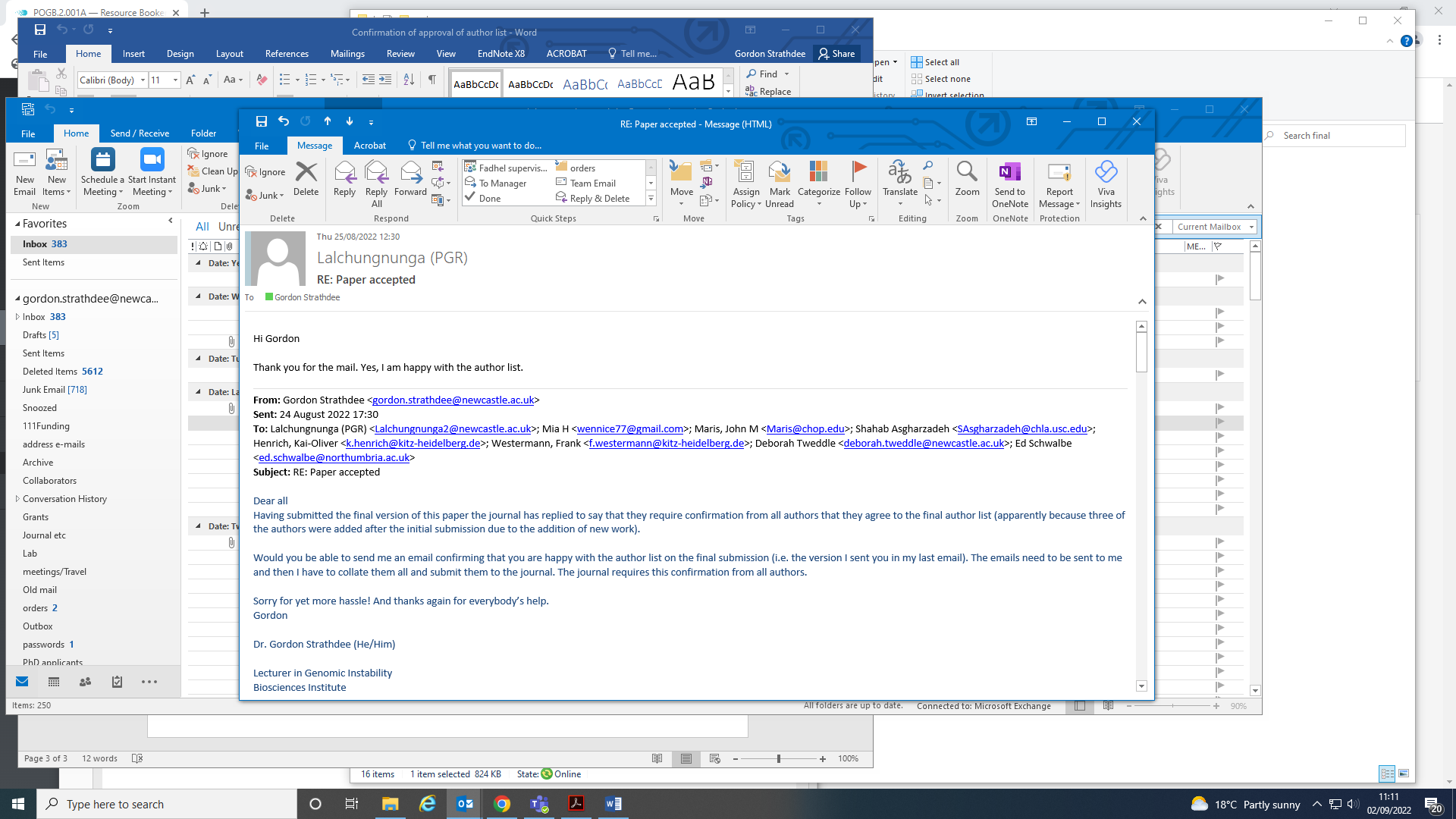


Email confirmation from Deborah Tweddle


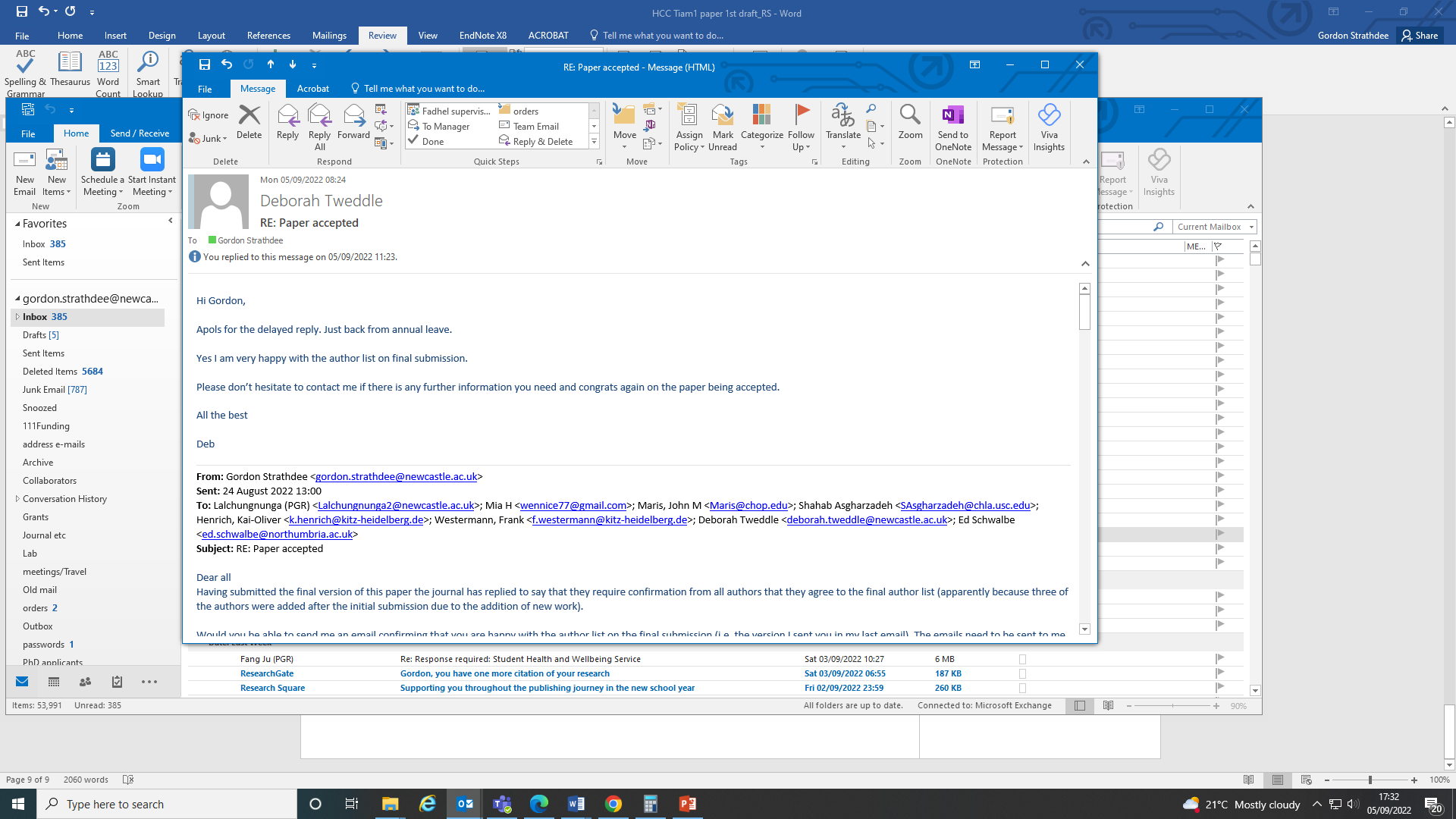


Email from Kai-Oliver Henrich


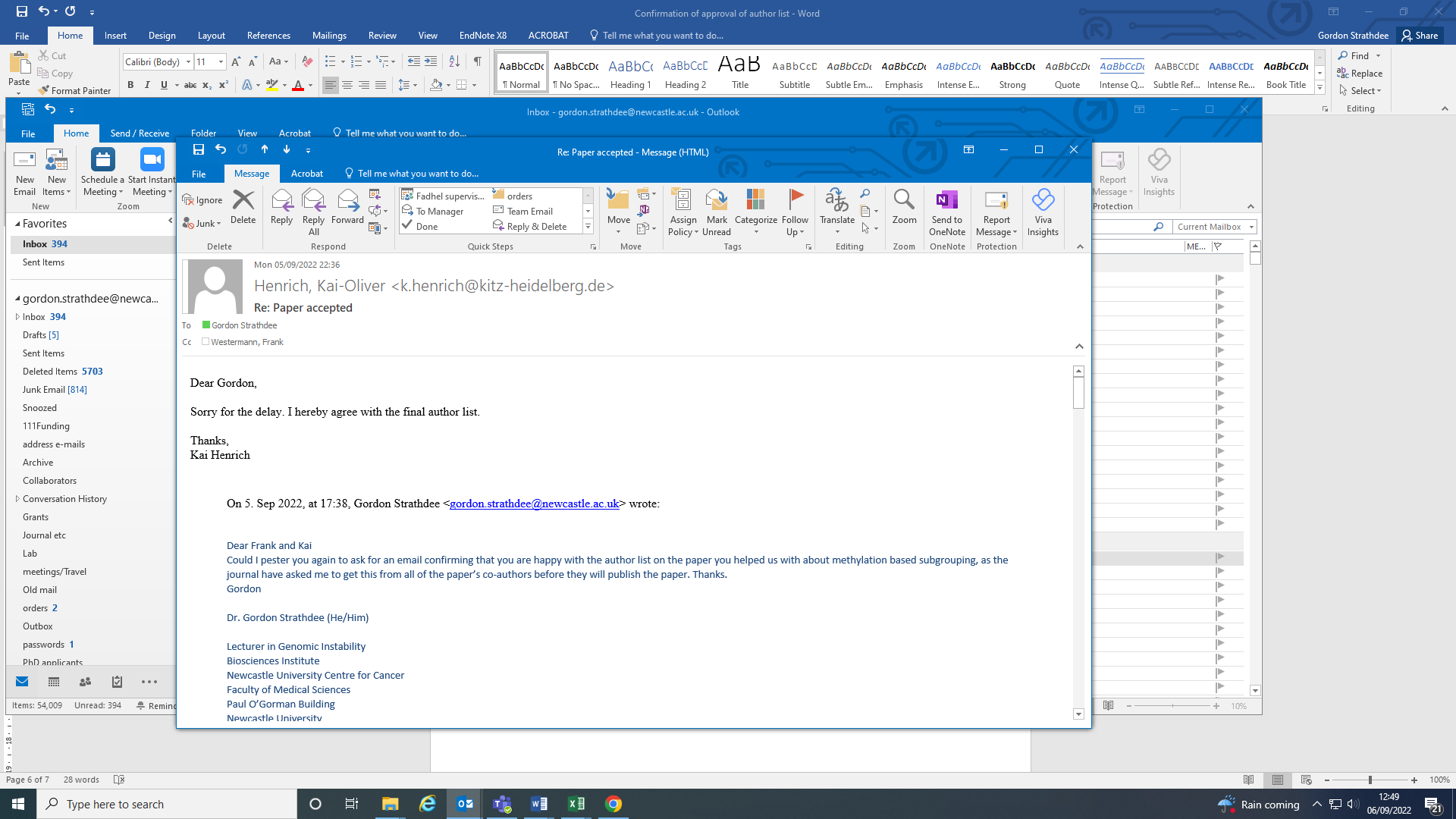


Email from Frank Westermann


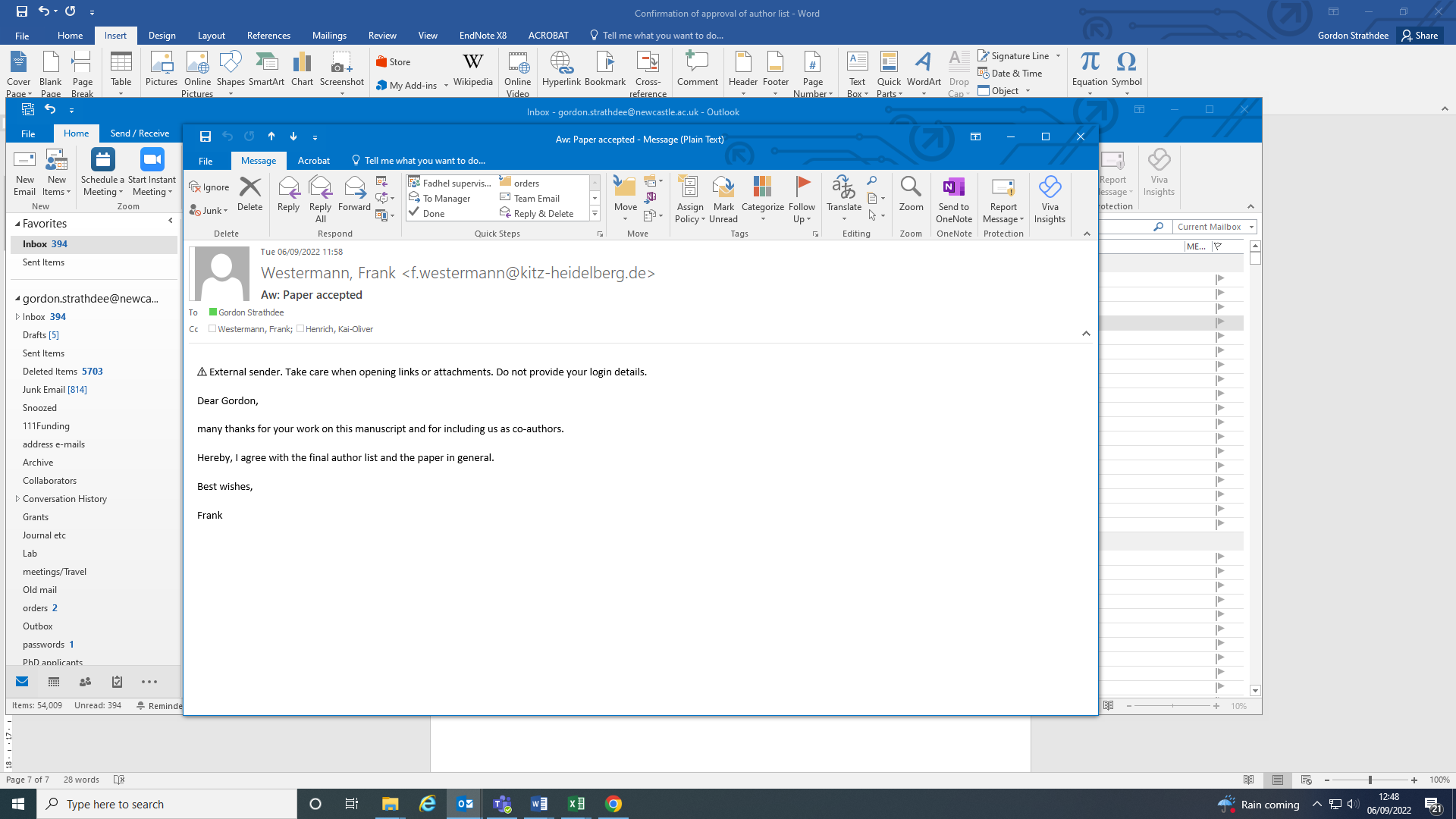


Email from Shahab Asgharzadeh:


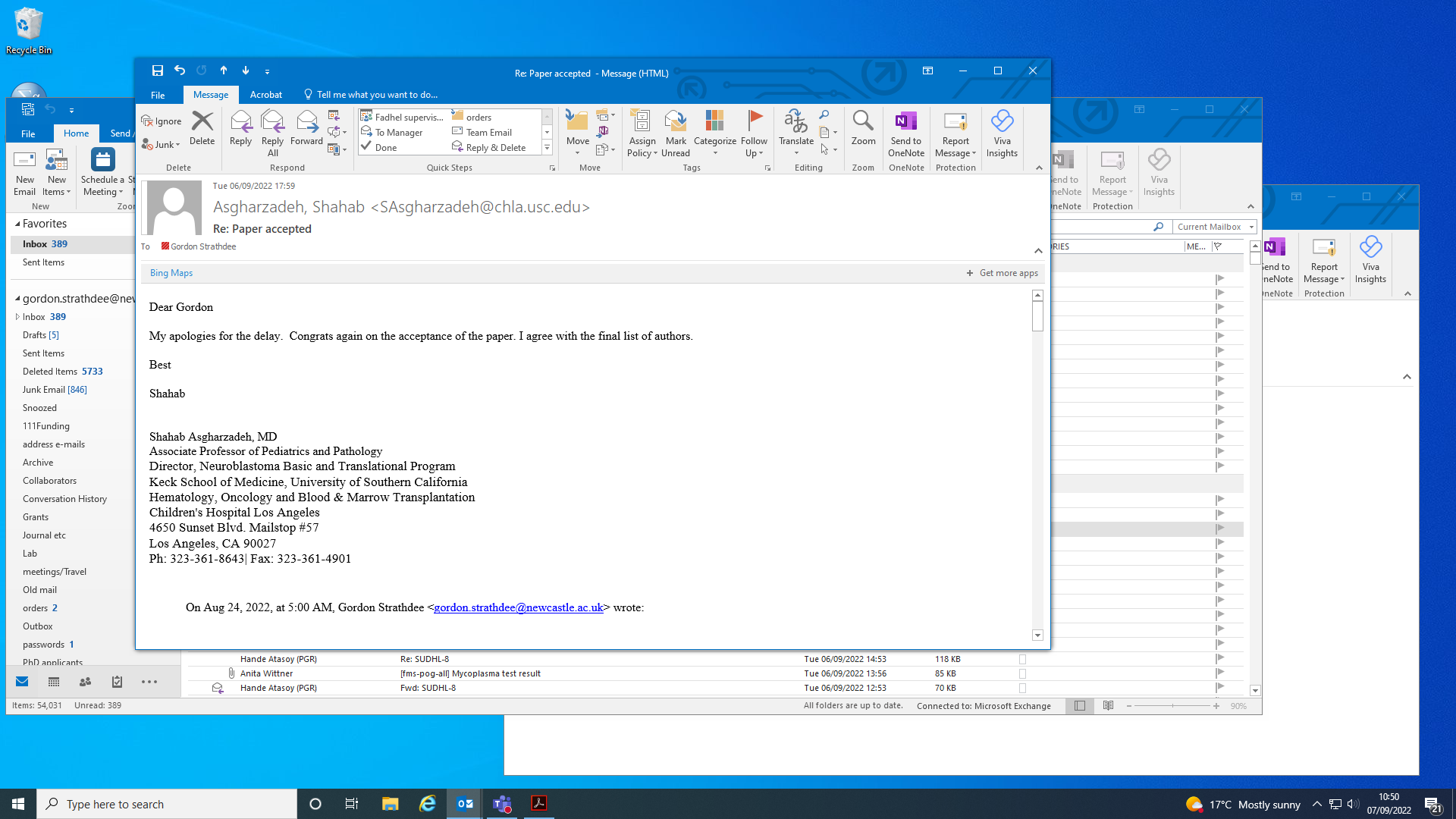

Supplement: Supplementary file 2 — Confirmation of approval of author list [file 41416_2022_1988_MOESM2_ESM.docx]
